# Beneficiary Experience of Care by Level of Integration in Dual Eligible Special Needs Plans

**DOI:** 10.1001/jamahealthforum.2024.1383

**Published:** 2024-06-07

**Authors:** Jennifer M. Mellor, Peter J. Cunningham, Erin Britton, Matthew Behrens, Atika Farzana Urmi, Valentina Vega

**Affiliations:** 1 Department of Economics, William & Mary, Williamsburg, Virginia; 2 Schroeder Center for Health Policy, William & Mary, Williamsburg, Virginia; 3 Department of Health Policy, Virginia Commonwealth University, Richmond; 4 Institute for Accountable Care, Brandeis University, Washington, DC; 5 Virginia Department of Medical Assistance Services, Richmond; 6 Department of Biostatistics, Virginia Commonwealth University, Richmond

## Abstract

**Importance:**

Dual Eligible Special Needs Plans (D-SNPs) are private managed care plans designed to promote Medicare and Medicaid integration for full-benefit, dually eligible beneficiaries. Currently, the highest level of D-SNP integration occurs in plans with exclusively aligned enrollment (EAE).

**Objective:**

To compare patient experience of care, out-of-pocket spending, and satisfaction among dually enrolled Medicaid beneficiaries in D-SNPs with EAE, those in D-SNPs without EAE, and those with traditional Medicare.

**Design, Setting, and Participants:**

This cross-sectional study included respondents to a mail survey fielded to a stratified random sample of full-benefit, community-dwelling, dual-eligible Medicaid beneficiaries who qualified for receipt of home and community-based services in the Virginia Medicaid Commonwealth Coordinated Care Plus program between March and October 2022.

**Exposure:**

Enrollment in a D-SNP with EAE or a D-SNP without EAE vs traditional Medicare.

**Main Outcomes and Measures:**

The main outcomes were self-reported measures of access and delays in receiving plan approvals, out-of-pocket spending, and satisfaction with health plans’ customer service and choice of primary care and specialist physicians.

**Results:**

Of 7200 surveys sent, 2226 were completed (response rate, 30.9%). The analytic sample consisted of 1913 Medicaid beneficiaries with nonmissing data on covariates (mean [SD] age, 70.8 [15.6] years; 1367 [71.5%] female). Of these, 583 (30.5%) were enrolled in D-SNPs with EAE, 757 (39.6%) in D-SNPs without EAE, and 573 (30.0%) in traditional Medicare. Compared with respondents enrolled in D-SNPs without EAE, those in D-SNPs with the highest level of integration (EAE) were 6.77 percentage points (95% CI, 8.81-12.66 percentage points) more likely to report being treated with courtesy and respect and 5.83 percentage points (95% CI, 0.21-11.46 percentage points) more likely to know who to call when they had a health problem. No statistically significant differences were found between members in either type of D-SNP and between those in D-SNPs and traditional Medicare in terms of their difficulty accessing care, delays in care, and satisfaction with care coordination and physician choice.

**Conclusions and Relevance:**

This cross-sectional study found some benefits of integrating administrative processes under Medicare and Medicaid but suggests that care coordination and access improvements under full integration require additional time and/or efforts to achieve.

## Key Points

**Question:**

Do dually enrolled Medicaid beneficiaries in highly integrated Dual Eligible Special Needs Plans (D-SNPs) have a better experience of care than beneficiaries enrolled in less integrated D-SNPs and Medicare fee-for-service?

**Findings:**

In this cross-sectional study of 1913 dually enrolled Medicaid beneficiaries who used home and community-based services in Virginia, members in more highly integrated D-SNPs were significantly more likely to report satisfaction with some plan dimensions compared with beneficiaries in less integrated D-SNPs. There were no significant differences related to access to care.

**Meaning:**

In this study, greater integration of Medicare and Medicaid benefits was associated with a few improved aspects of patient experience of care.

## Teaser

This cross-sectional study compares experience of care, out-of-pocket spending, and satisfaction between dually enrolled Medicaid beneficiaries with traditional Medicare and those with Dual Eligible Special Needs Plans with and without exclusively aligned enrollment.

## Introduction

Compared with Medicare beneficiaries without Medicaid, dually eligible beneficiaries have higher rates of chronic illness, more activity of daily living (ADL) limitations, and fewer social supports.^1^ Yet, most navigate Medicare and Medicaid as separate programs with different coverage rules, physician networks, and administrative processes, which may lessen their experience of care and contribute to administrative burden.^2,3,4,5,6^ To reduce care fragmentation for this vulnerable group, states and the federal government have taken steps to integrate Medicare and Medicaid. One means of promoting Medicare-Medicaid integration is through Dual Eligible Special Needs Plans (D-SNPs), which are private Medicare Advantage (MA) plans that serve only dually eligible beneficiaries under a contract with both the Centers for Medicare & Medicaid Services (CMS) and the state Medicaid plan. In 2020, 32% of the nation’s 12.9 million dually enrolled beneficiaries were enrolled in D-SNPs.^7^

D-SNPs range from coordination-only plans to highly integrated (HIDE) and fully integrated (FIDE) plans. The highest level of integration occurs in HIDE or FIDE SNPs with exclusively aligned enrollment (EAE), hereafter called D-SNPs with EAE.^4,7^ Under aligned enrollment, a beneficiary’s D-SNP and Medicaid managed care plan are offered by the same parent company. Under exclusively aligned enrollment, enrollment in a D-SNP is limited to only members who have aligned enrollment. Aligned enrollment may reduce service duplication in Medicare and Medicaid, while EAE creates unified coverage with a single network, grievance process, and customer service system that may improve access to care, enhance care coordination, and allow plans to tailor enhanced benefits to members’ needs.^8^ In 2022, D-SNPs with EAE enrolled fewer than 5% of dually eligible beneficiaries.^4,8^ In 2025, CMS will require FIDE SNPs to operate with EAE.^9^

Despite these developments, there is limited evidence on the benefits of D-SNPs. Prior studies reported that members in D-SNPs experienced similar or better access to and satisfaction with care as those in traditional Medicare but were more likely to have emergency department visits compared with dually eligible beneficiaries in non-D-SNP MA plans.^10,11,12^ In counties with higher proportions of dually eligible beneficiaries in aligned D-SNPs, dually eligible beneficiaries were less likely to use inpatient care and nursing facility care and had fewer prescriptions.^13^ To our knowledge, no prior study has compared outcomes for members in D-SNPs with and without EAE.

Analysis of state-level changes can offer important insights given the lack of research and pending regulatory changes in this area. Virginia, 1 of 9 states offering D-SNPs with EAE, experienced a recent change in EAE enrollment.^8^ Since 2017, dually eligible beneficiaries have been enrolled in a managed care program for individuals aged 65 years or older and people with disabilities (called Commonwealth Coordinated Care Plus [CCC Plus]). Virginia requires that all Medicaid managed care organizations (MCOs) offer a D-SNP in Virginia and that all D-SNPs offer a FIDE SNP. While Virginia does not currently require that FIDE SNPs use exclusively aligned enrollment, several insurers chose to transition previously aligned members to new D-SNPs with EAE in 2021. By 2022, 3 of the 10 D-SNPs serving full-benefit, dually eligible beneficiaries in Virginia were FIDE SNPs with EAE, with some insurers offering D-SNPs with and without EAE.

In this study, we used the insurers’ programmatic change combined with a survey of dually enrolled Virginia Medicaid beneficiaries to assess how access to care, out-of-pocket spending, and satisfaction differed between members enrolled in D-SNPs with EAE, those in D-SNPs without EAE, and those with traditional Medicare. The study population was limited to persons enrolled in Virginia’s 1915(c) waiver for home and community-based services (HCBS); thus, our analysis focused on people with frailty and/or disability who could greatly benefit from integration.

## Methods

This cross-sectional study followed the Strengthening the Reporting of Observational Studies in Epidemiology (STROBE) reporting guideline. The study was approved by the Virginia Commonwealth University institutional review board. As approved by the institutional review board, informed consent was obtained through return of the completed survey questionnaire.

### Data

We surveyed dually enrolled Virginia Medicaid members using a stratified random sampling design. The sample frame consisted of community-dwelling, full-benefit, dually eligible beneficiaries enrolled in Virginia’s CCC Plus program and 1915(c) waiver program for HCBS. We constructed 3 strata based on enrollment in (1) traditional Medicare, (2) D-SNPs with EAE, and (3) D-SNPs without EAE (ie, HIDE and FIDE plans with either a mix of aligned and unaligned members or unaligned members only). We excluded dually enrolled members in non–D-SNP MA plans since these plans accounted for fewer than 10% of Virginia dual beneficiaries. Inclusion criteria for all strata specified that members be enrolled for at least 6 months in (1) full-benefit Medicaid, (2) the same Medicaid MCO, and (3) the state’s HCBS waiver program. For members in D-SNPs with EAE, eligibility was also restricted to members in their plan for at least 6 months since the plan moved to EAE. All inclusion criteria were applied using Virginia Medicaid administrative enrollment data. Once the strata were constructed, a total of 7200 survey participants were selected through simple random sampling within each stratum.

Questionnaires and other survey materials were mailed in 3 waves between March and October 2022. The questionnaire was constructed by adapting questions from prior and ongoing surveys, including a survey conducted as part of the Cal MediConnect demonstration, the Care Coordination Measure for Primary Care survey from the Agency for Healthcare Research and Quality, and the MA Consumer Assessment of Healthcare Providers and Systems questionnaire by CMS.^14,15,16^ The instrument also included questions from prior surveys of Virginia Medicaid members. A mail survey was used primarily because postal addresses were the best source of contact information in the enrollment files (there were no email addresses and, in most cases, no telephone numbers). To increase response rates, a $5 incentive was included in the mailing, and there were 2 additional follow-up mailings to sample persons who did not initially respond. Survey materials mailed to respondents included a letter explaining the purpose of the study, that their participation was strictly voluntary, and that their individual responses would not be shared with Virginia Medicaid.

We assessed potential nonresponse bias using Medicaid enrollment data to compare survey respondents with the total sample on age, sex, race, ethnicity, and rural residence (eAppendix 1 and eTable 1 in Supplement 1). With some exceptions, respondents were generally comparable to the total sample on these characteristics, suggesting that the sample was not overly biased. In addition, the propensity score weighting methods used in our analysis may have further reduced nonresponse bias. Nevertheless, other unknown differences between respondents and nonrespondents may have remained and been unaccounted for in the analysis.

### Outcomes

To measure access to care, we defined binary measures indicating whether respondents reported at least some difficulty with 9 types of care (primary care, specialist care, mental health services, prescription medications, medical equipment and/or supplies, dental, vision, hearing care, and HCBS) and an indicator for whether they reported at least some difficulty with any type of care relative to no difficulty or not needing this type of care. We also defined 3 binary measures of whether the member experienced delays in approval for medications, specialists, or HCBS. To measure out-of-pocket spending, we included total out-of-pocket spending in the past 6 months, indicators for types of out-of-pocket costs (eg, health care practitioner visits, prescription drugs, dental care, vision care, and medical equipment), and a measure of whether out-of-pocket costs were a major financial burden. To measure member satisfaction, we included binary indicators of whether the member was satisfied with their health plan’s care coordinator, choice of both primary care and specialist physicians, and customer service and the member’s overall rating of their health plan. We also examined whether respondents agreed with statements indicating that they knew who to call with questions about their health or health care, felt confident in their understanding of the health care system. and found caring for their health and chronic conditions manageable.

### Explanatory Variables of Interest and Covariates

In the first model, we included 2 explanatory variables of interest: indicators for enrollment in a D-SNP with EAE and enrollment in a D-SNP without EAE compared with traditional Medicare. In a second model, we included a single D-SNP indicator compared with traditional Medicare. Both models controlled for members’ age, sex, educational level, marital status, number of ADL limitations, and whether they had ever been diagnosed with any of 11 health conditions as defined from survey responses. We also controlled for race and ethnicity given evidence that D-SNP enrollment is higher among Black and Hispanic Medicaid members.^11^ Our survey used separate questions for Hispanic ethnicity and race, and members of each racial group included Hispanic and non-Hispanic persons. We separately defined an indicator for Hispanic ethnicity and indicators of whether members identified as Black, White, or other racial group (specifically Asian, Native American, >1 race, or race not indicated). To control for residence in a rural or urban area, we linked the respondent’s mailing address zip code to 2010 rural-urban commuting area codes.

### Statistical Analysis

We estimated linear probability regression models for each outcome. To reduce the potential that nonrandom selection into types of Medicare coverage biased our results, we weighted observations by the inverse probability of treatment (ie, propensity score weights) estimated from a model of Medicare plan type using respondent observable traits as explanatory variables as detailed in eAppendix 2 and eFigures 1 and 2 in Supplement 1; this adjusted for observable differences among people with different types of Medicare coverage. To assess statistically significant differences in outcomes by Medicare coverage type, we used *t* tests and discerned significance using 2-sided *P* < .05. In sensitivity analysis, we estimated models using alternate definitions of outcomes pertaining to difficulty with care and models of difficulty conditional on needing a particular type of care. Stata, version 15.1 (StataCorp LLC) was used for analysis.

## Results

### Study Sample

Of 7200 surveys sent, we received 2226 completed surveys, for a response rate of 30.9%. Of members who returned surveys, 651 (29.2%) were in traditional Medicare, 696 (31.3%) were in FIDE SNPs with EAE, and 879 (39.5%) were enrolled in D-SNPs without EAE. The analytic sample consisted of 1913 Medicaid HCBS users with nonmissing data on covariates (mean [SD] age, 70.8 [15.6] years; 1367 [71.5%] female; 546 [28.5%] male), including 573 (30.0%) respondents with traditional Medicare, 583 (30.5%) in D-SNPs with EAE, and 757 (39.6%) in D-SNPs without EAE (Table 1). In the total analytic sample, 838 (43.8%) were Black, 48 (2.5%) were Hispanic, 851 (44.5%) were White, and 224 (11.7%) were other race. Respondents in D-SNPs were 3.16 years younger and had 0.42 fewer ADL limitations than respondents with traditional Medicare. Respondents in D-SNPs were more likely to report Black race than respondents in traditional Medicare. When we weighted by propensity score weights, we observed no statistically significant differences in demographic characteristics, number of ADL limitations, and clinical diagnoses (eAppendix 3 and eTable 2 in Supplement 1).

**Table 1.** Demographics, ADLs, and Diagnosed Clinical Conditions Among Dually Eligible Users of Home and Community-Based Services by Medicare Plan Type

Characteristic	Beneficiaries^a^				*P* value, vs traditional Medicare^b^	
	Total (N = 1913)	Traditional Medicare (n = 573)	D-SNPs with EAE (n = 583)	D-SNPs without EAE (n = 757)	D-SNP with EAE	D-SNP without EAE
Age, mean (SD), y	70.80 (15.58)	72.93 (17.32)	69.77 (14.04)	69.98 (15.18)	.001	.001
Sex						
Female	1367 (71.5)	421 (73.5)	401 (68.8)	545 (72)	.08	.55
Male	546 (28.5)	152 (26.5)	182 (31.2)	212 (28.0)		
Race and ethnicity						
Black	838 (43.8)	198 (34.6)	257 (44.1)	383 (50.6)	.001	<.001
Hispanic	224 (11.7)	123 (21.5)	31 (5.3)	70 (9.2)	.17	.78
White (reference)	851 (44.5)	252 (43.9)	295 (50.6)	304 (40.2)	NA	NA
Other^c^	48 (2.5)	16 (2.8)	9 (1.5)	23 (3.0)	.001	<.001
Educational level						
More than high school (reference)	519 (27.1)	148 (25.8)	168 (28.8)	203 (26.8)	NA	NA
High school	601 (31.4)	176 (30.7)	200 (34.3)	225 (29.7)	.19	.70
Less than high school	793 (41.5)	249 (43.5)	215 (36.9)	329 (43.5)	.02	.99
Marital status						
Married (reference)	315 (16.5)	95 (16.8)	98 (16.8)	121 (16.0)	NA	NA
Never married	437 (22.8)	152 (26.5)	109 (18.7)	176 (23.2)	.002	.16
Separated or divorced	532 (27.8)	104 (18.2)	198 (34.0)	230 (30.4)	.001	<.001
Widowed	629 (32.9)	221 (38.6)	178 (30.5)	230 (30.4)	.004	.002
Rural resident^d^	527 (27.5)	129 (22.5)	178 (30.5)	220 (29.1)	.002	.01
ADL limitations, mean (SD), No.^e^	3.67 (2.00)	3.95 (1.99)	3.53 (1.99)	3.58 (2.01)	<.001	.001
Diagnosed health conditions						
Heart condition	854 (44.6)	260 (45.4)	262 (44.9)	332 (43.9)	.88	.58
Stroke	477 (24.9)	142 (24.8)	150 (25.7)	185 (24.4)	.71	.89
COPD	484 (25.3)	106 (18.5)	173 (29.7)	205 (27.1)	<.001	<.001
Cancer	347 (18.1)	108 (18.8)	110 (18.9)	129 (17.0)	.99	.40
Diabetes	824 (43.1)	227 (39.6)	257 (44.1)	340 (44.9)	.13	.05
Asthma	518 (27.1)	152 (26.5)	155 (26.6)	211 (27.9)	.98	.58
Intellectual or developmental disability	335 (17.5)	117 (20.4)	83 (14.2)	135 (17.8)	.006	.22
Depression or anxiety	872 (45.8)	251 (43.8)	257 (44.1)	364 (48.1)	.92	.12
Dementia	354 (18.5)	131 (22.9)	96 (16.5)	127 (16.8)	.005	.005
Substance use disorder	68 (3.6)	20 (3.5)	19 (3.3)	29 (3.8)	.83	.74
Another chronic disease	338 (17.7)	95 (16.6)	116 (19.9)	127 (16.8)	.14	.93

Abbreviations: ADL, activity of daily living; COPD, chronic obstructive pulmonary disease; D-SNPs, Dual Eligible Special Needs Plans; EAE, exclusively aligned enrollment; NA, not applicable.

^a^ Data are presented as number (percentage) of beneficiaries unless otherwise indicated.

^b^ Tests of significant differences in the proportion of respondents with each characteristic (or variable mean in the case of the ADL limitation count) among dually eligible beneficiaries in each type of D-SNP compared with dually eligible beneficiaries enrolled in traditional Medicare. Estimates are based on 1913 observations with complete data on the respondent characteristics.

^c^ Included persons who identified as Asian, Native American, more than 1 race, or race not indicated.

^d^ Residence in a rural area based on rural-urban commuting area codes developed by the US Department of Agriculture.

^e^ Number of ADLs that a respondent reported difficulty performing alone and without using special equipment.

### Access to Care

Compared with respondents in traditional Medicare, those in D-SNPs with EAE did not significantly differ in terms of difficulty accessing the 9 specific types of services (Table 2; eAppendix 5 and eTable 5 in Supplement 1 give more information on the outcome variable construction, and eAppendix 6 and eTables 6-11 in Supplement 1 give the full model results). Similarly, respondents in D-SNPs without EAE did not significantly differ from those enrolled in traditional Medicare in difficulty accessing services. We observed no significant differences in access difficulties by level of D-SNP integration. Reports of delays were not significantly different among respondents in either type of D-SNP compared with respondents in traditional Medicare, and we observed no differences in delays by the level of D-SNP integration. We obtained similar results using alternate definitions and samples for outcomes pertaining to access difficulties and when we counted the number of access difficulties (eAppendix 4 and eTables 3 and 4 in Supplement 1).

**Table 2.** Access to Care Overall and Differences by Type of Medicare Plan Among Dually Eligible Users of HCBS^a^

Outcome	Beneficiaries, mean (SD), %	D-SNPs with EAE vs traditional Medicare		D-SNPs without EAE vs traditional Medicare		D-SNPs with EAE vs without EAE		All D-SNPs vs traditional Medicare	
		Difference, PPs (95% CI)^b^	*P* value^c^	Difference, PPs (95% CI)^b^	*P* value^c^	Difference, PPs (95% CI)^b^	*P* value^c^	Difference, PPs (95% CI)^b^	*P* value^c^
**Difficulty** ^d^									
Primary care	10.9 (31.1)	−2.55 (−6.34 to 1.23)	.19	−1.16 (−4.73 to 2.40)	.52	−1.39 (−4.87 to 2.09)	.78	−1.88 (−5.06 to 1.29)	.25
Specialist care	13.8 (34.5)	−0.15 (−4.49 to 4.19)	.95	−1.90 (−5.71 to 1.90)	.33	1.75 (−2.24 to 5.74)	.39	−1.14 (−4.61 to 2.33)	.52
Mental health services	5.6 (23.1)	−0.49 (−3.34 to 2.37)	.74	−0.17 (−2.82 to 2.48)	.90	−0.31 (−2.96 to 2.33)	.82	−0.63 (−2.98 to 1.72)	.60
Prescription medications	12.5 (33.1)	−2.37 (−6.61 to 1.88)	.27	−3.31 (−7.12 to 0.51)	.09	0.94 (−2.79 to 4.67)	.62	−3.06 (−6.55 to 0.44)	.09
Medical equipment and/or supplies	18.5 (38.9)	−3.37 (−8.20 to 1.46)	.17	−3.17 (−7.66 to 1.32)	.17	−0.20 (−4.51 to 4.11)	.93	−3.72 (−7.78 to 0.35)	.07
Dental care	20.5 (40.4)	−1.45 (−6.35 to 3.46)	.56	−1.12 (−5.59 to 3.36)	.62	−0.33 (−4.94 to 4.27)	.88	−1.58 (−5.60 to 2.43)	.44
Vision care	12.7 33.3)	−1.02 (−5.20 to 3.17)	.63	−1.03 (−4.79 to 2.73)	.59	0.01 (−3.84 to 3.87)	.99	−1.27 (−4.66 to 2.12)	.46
Hearing care	9.3 (29.1)	−0.76 (−4.29 to 2.78)	.68	−2.48 (−5.56 to 0.59)	.11	1.73 (−1.64 to 5.09)	.31	−2.04 (−4.83 to 0.74)	.15
HCBS^e^	16.9 (37.4)	−0.30 (−4.89 to 4.29)	.90	2.31 (−1.98 to 6.61)	.29	−2.61 (−7.01 to 1.78)	.24	1.29 (−2.54 to 5.13)	.51
Any care	46.2 (49.9)	−5.30 (−11.26 to 0.67)	.08	−1.19 (−6.75 to 4.37)	.67	−4.11 (−9.63 to 1.42)	.15	−2.81 (−7.81 to 2.19)	.27
**Delays in receiving plan approvals** ^f^									
Medications	31.4 (46.4)	−1.60 (−7.37 to 4.16)	.59	0.79 (−4.59 to 6.17)	.77	−2.40 (−7.64 to 2.86)	.37	−0.20 (−5.07 to 4.66)	.93
Specialists	27.3 (44.5)	1.70 (−3.82 to 7.21)	.55	5.01 (−0.13 to 10.15)	.06	−3.32 (−8.50 to 1.87)	.21	3.53 (−1.09 to 8.15)	.13
HCBS	32.2 (46.7)	0.61 (−5.21 to 6.43)	.84	2.31 (−3.22 to 7.83)	.41	−1.70 (−7.02 to 3.63)	.53	1.59 (−3.38 to 6.56)	.53

Abbreviations: D-SNPs, Dual Eligible Special Needs Plans; EAE, exclusively aligned enrollment; HCBS, home and community-based services; PPs, percentage points.

^a^ eAppendix 5 in Supplement 1 gives more information on the outcome variable construction, and eAppendix 6 in Supplement 1 gives the full model results.

^b^ Estimates were adjusted for the covariates in Table 1 and were weighted using propensity score weights.

^c^ Significance levels based on robust SEs.

^d^ Defined as having a lot of difficulty or some difficulty vs no difficulty or no need.

^e^ Defined in the survey as “care and other in-home services and conveniences that help with daily activities (personal care services, adult day care, skilled nursing, etc).”

^f^ Text of questions refers to delays in getting plan approvals from the respondent’s Medicaid health plan.

### Out-of-Pocket Spending

Mean (SD) out-of-pocket costs were $227.24 ($1074.01), and out-of-pocket costs were less for respondents in D-SNP with EAE (−54.4%; 95% CI, −68.5% to −34.2%) and those in D-SNPs without EAE (−56.0%; 95% CI, −68.6% to −38.1%) compared with those in traditional Medicare (Table 3). Respondents in D-SNPs were less likely to have out-of-pocket costs for prescription drugs, dental care, vision care, and medical equipment compared with those in traditional Medicare. Among dually eligible beneficiaries in D-SNPs, we did not observe significant differences in total out-of-pocket spending by level of integration; however, dually eligible beneficiaries in more integrated D-SNPs were less likely to report out-of-pocket costs for vision care (−2.85 percentage points; 95% CI, −5.44 to −0.26 percentage points; *P* = .03) compared with those in less integrated D-SNPs. Overall, 267 dually enrolled beneficiaries (14.9%) reported that out-of-pocket costs were a major financial burden. Compared with respondents in traditional Medicare, those in more integrated D-SNPs were less likely to report major financial burden (−6.35 percentage points; 95% CI, −10.86 to –1.83 percentage points), but there was no difference for those in other D-SNPs (−3.73 percentage points; 95% CI, −8.02 to 0.55 percentage points).

**Table 3.** Out-of-Pocket Spending Overall and Differences by Type of Medicare Plan Among Dually Eligible Users of Home and Community-Based Services^a^

Outcome	Beneficiaries, mean (SD) %	D-SNPs with EAE vs traditional Medicare		D-SNPs without EAE vs traditional Medicare		D-SNPs with EAE vs D-SNPs without EAE		All D-SNPs vs traditional Medicare	
		Difference, PPs (95% CI)^b^	*P* value^c^	Difference, PPs (95% CI)^b^	*P* value^c^	Difference, PPs (95% CI)^b^	*P* value^c^	Difference, PPs (95% CI)^b^	*P* value^c^
OOP spending, $	227.24 (1074.01)	−54.4 (−68.5 to −34.2)^d^	<.001	−56.0 (−68.6 to −38.1)^d^	<.001	3.4 (−24.6 to 41.8)^d^	.84	−56.3 (−68.0 to −40.4)^d^	<.001
Any OOP spending									
Overall	44.5 (49.7)	−11.73 (−18.03 to −5.44)	<.001	−11.45 (−17.22 to −5.67)	<.001	−0.29 (−5.98 to 5.40)	.92	−11.94 (−17.17 to −6.71)	<.001
Physician visits	8.2 (27.5)	1.43 (−2.12 to 4.98)	.43	1.45 (−1.86 to 4.76)	.39	−0.02 (−3.26 to 3.22)	.99	1.67 (−1.36 to 4.69)	.28
Prescription drugs	14.2 (34.9)	−6.23 (−10.71 to −1.74)	.01	−4.43 (−8.78 to −0.08)	.05	−1.79 (−5.56 to 1.97)	.35	−5.22 (−9.19 to −1.26)	.01
Dental care	7.8 (26.9)	−6.84 (−10.26 to −3.41)	<.001	−4.55 (−7.92 to −1.18)	.01	−2.29 (−5.02 to 0.44)	.10	−5.47 (−8.54 to −2.40)	<.001
Vision care	7.1 (25.6)	−3.60 (−6.64 to −0.56)	.02	−0.75 (−3.95 to 2.46)	.65	−2.85 (−5.44 to −0.26)	.03	−1.79 (−4.67 to 1.08)	.22
Medical equipment	9.2 (28.9)	−4.50 (−8.11 to −0.89)	.01	−3.27 (−6.73 to 0.18)	.06	−1.23 (−4.37 to 1.91)	.44	−3.99 (−7.10 to −0.88)	.01
Feel OOP costs are a major financial burden	14.9 (35.6)	−6.35 (−10.86 to –1.83)	.01	−3.73 (−8.02 to 0.55)	.09	−2.61 (−6.58 to 1.36)	.20	−4.87 (−8.75 to –0.99)	.01

Abbreviations: D-SNPs, Dual Eligible Special Needs Plans; EAE, exclusively aligned enrollment; OOP, out-of-pocket; PPs, percentage points.

^a^ eAppendix 5 in Supplement 1 gives more information on the outcome variable construction, and eAppendix 6 in Supplement 1 gives the full model results.

^b^ Estimates were adjusted for the covariates in Table 1 and were weighted using propensity score weights.

^c^ Significance levels based on robust SEs.

^d^ Percentage difference.

### Satisfaction

Most dually enrolled beneficiaries were very satisfied with their plans’ care coordinator and choice of primary care and specialist physicians, and there were no differences in these dimensions of satisfaction by Medicare type (Table 4). Compared with respondents in traditional Medicare, respondents in more integrated D-SNPs were 6.88 percentage points (95% CI, 0.56-13.20 percentage points) more likely to report that customer service always treated them respectfully and were 8.54 percentage points (95% CI, 2.49-14.58 percentage points) more likely to give their plan the highest rating. Compared with respondents in less integrated plans, respondents in more integrated D-SNPs were 6.77 percentage points (95% CI, 8.81-12.66 percentage points) more likely to report that customer service always treated them respectfully and 5.83 percentage points (95% CI, 0.21-11.46 percentage points) more likely to know how to get information about health and health care.

**Table 4.** Satisfaction Overall and Differences by Type of Medicare Plan Among Dually Eligible Users of Home and Community-Based Services^a^

Outcome	Beneficiaries, mean (SD), %	D-SNPs with EAE vs traditional Medicare		D-SNPs without EAE vs traditional Medicare		D-SNPs with EAE vs D-SNPs without EAE		All D-SNPs vs traditional Medicare	
		Difference, PPs (95% CI)^b^	*P* value^c^	Difference, PPs (95% CI)^b^	*P* value^c^	Difference, PPs (95% CI)^b^	*P* value^c^	Difference, PPs (95% CI)^b^	*P* value^c^
Very satisfied^d^									
Care coordinator	67.5 (46.8)	−2.15 (−8.25 to 3.95)	.49	1.19 (−4.42 to 6.80)	.68	−3.34 (−8.94 to 2.26)	.24	−0.28 (−5.36 to 4.80)	.92
PCP choice	60.1 (50.0)	1.47 (−4.76 to 7.70)	.64	−0.07 (−5.81 to 5.68)	.98	1.54 (−4.09 to 7.17)	.59	0.70 (−4.52 to 5.91)	.79
Specialist choice	56.4 (49.6)	0.22 (−6.12 to 6.57)	.95	−3.49 (−9.32 to 2.33)	.24	3.72 (−2.06 to 9.49)	.21	−1.77 (−7.06 to 3.52)	.51
Customer service^d^									
Always gives needed information or help	45.7 (49.8)	4.49 (−3.51 to 12.49)	.27	1.31 (−6.52 to 9.13)	.74	3.18 (−4.03 to 10.40)	.39	3.54 (−3.39 to 10.47)	.32
Always treats with courtesy and respect	78.3 (41.2)	6.88 (0.56 to 13.20)	.03	0.11 (−6.36 to 6.58)	.97	6.77 (8.81 to 12.66)	.02	3.23 (−2.37 to 8.83)	.26
Plan rating, mean (SD)^e^	8.68 (1.7)	0.29 (0.08 to 0.51)^f^	.01	0.12 (−0.08. to 0.32)^f^	.26	0.18 (−0.01 to 0.37)^f^	.07	0.20 (0.02 to 0.38)^f^	.03
Rated plan a 10^d^	45.4 (49.8)	8.54 (2.49 to 14.58)	.01	3.80 (−1.84 to 9.44)	.19	4.74 (0.78 to 10.25)	.09	5.92 (0.84 to 11.00)	.02
Strongly agree									
Know who to call about health or health care	46.0 (49.9)	4.84 (−1.35 to 11.03)	.13	−0.99 (−6.78 to 4.79)	.74	5.83 (0.21 to 11.46)	.04	1.34 (−3.88 to 6.56)	.61
Confident in understanding of health care system	34.0 (47.4)	5.38 (−0.39 to 11.15)	.07	2.79 (−2.61 to 8.20)	.31	2.59 (−2.72 to 7.89)	.34	3.92 (−0.92 to 8.77)	.11
Caring for health and chronic conditions is manageable	32.7 (46.9)	5.29 (−0.52 to 11.10)	.07	2.56 (−2.85 to 7.98)	.35	2.72 (−2.58 to 8.03)	.31	3.76 (−1.14 to 8.65)	.13

Abbreviations: D-SNPs, Dual Eligible Special Needs Plans; EAE, exclusively aligned enrollment; PCP, primary care physician; PPs, percentage points.

^a^ eAppendix 5 in Supplement 1 gives more information on the outcome variable construction, and eAppendix 6 in Supplement 1 gives the full model results.

^b^ Estimates were adjusted for the covariates in Table 1 and were weighted using propensity score weights.

^c^ Significance levels based on robust SEs.

^d^ Question text refers to respondent’s Medicaid health plan.

^e^ Scale is from 1 to 10.

^f^ Unit differences.

## Discussion

Dually eligible beneficiaries represent an important segment of both Medicare and Medicaid members. Average spending by full-benefit, dually eligible members is 2.5 times that of Medicare-only beneficiaries and 3.7 times that of Medicaid-only beneficiaries.^17^ To improve beneficiary health and care coordination, states and the federal government have sought to integrate Medicare and Medicaid through federal regulations, legislative proposals, and state contracting requirements for MCOs.

Virginia Medicaid has taken various steps to increase integration, including implementing mandatory statewide Medicaid managed care for older individuals and people with disabilities, using intelligent assignment (in which members’ initial Medicaid MCO assignment may be the MCO affiliated with their most recent MA plan) and default enrollment (in which D-SNPs can enroll members aging into Medicare from an affiliated Medicaid MCO), and using D-SNP contracts to encourage EAE. In this study, we provided early evidence on the impacts of increased integration from a survey of dually enrolled beneficiaries who used HCBS in Virginia Medicaid.

First, compared with respondents in traditional Medicare, dually eligible beneficiaries in D-SNPs had lower total out-of-pocket spending and those in D-SNPs with EAEs were less likely to report that out-of-pocket spending was a major burden. Because we observed differences in respondent reports of out-of-pocket spending for prescription drugs, dental, and vision care (services not covered by traditional Medicare), we attributed these differences in total out-of-pocket spending to the more generous benefits that MA plans offer. Other studies found that persons enrolled in D-SNPs reported higher satisfaction with out-of-pocket spending than persons in non–D-SNP MA plans.^11^

Second, there was some evidence of greater respondent satisfaction among members in more integrated D-SNPs compared with those in less integrated D-SNPs and traditional Medicare. These differences were observed in customer service interactions and overall plan ratings, which may be explained by the more unified administrative processes available in D-SNPs with EAE. We did not observe these differences in satisfaction among members in D-SNPs without EAE compared with those in traditional Medicare.

Third, while members in more integrated D-SNPs had a lower risk of vision out-of-pocket costs compared with members in less integrated D-SNPs, we found no differences by level of D-SNP integration in difficulty accessing care, delays in care, satisfaction with care coordination and physician networks, and total out-of-pocket spending. Some members in less integrated D-SNPs may have had aligned coverage, which would lessen differences between D-SNPs with and without EAE. Finally, we found few differences in experience of care and care coordination between dually eligible beneficiaries in D-SNPs compared with those in traditional Medicare. Both findings are notable because the study population consisted of HCBS users, a group that may have the greatest potential to benefit from integration. This may reflect the use of care coordination within Virginia Medicaid’s CCC Plus program and that most respondents reported being very satisfied with the Medicaid health plan's care coordination. However, we observed no differences in delays in receiving Medicaid plan approvals among members in D-SNPs compared with those in traditional Medicare. Since 863 members reported at least 1 type of delay, there remain opportunities to address this through plan integration.

Exclusively aligned enrollment is an important step in the evolution of integrated care for dually eligible beneficiaries, as evidenced by 2022 CMS rules that require all FIDE SNPs to operate with EAE starting in 2025.^9^ Our results provide insights into the benefits of EAE by drawing on early experiences in Virginia, where there have been considerable increases in Medicare-Medicaid integration since first contracting with D-SNPs in 2017. Additional integration efforts by states include plan model-of-care requirements and rules about how rebates achieved through MA plan bids are used to provide supplemental benefits.^18^ In January 2025, Virginia will transition to a D-SNP contract in which all D-SNP offerings have EAE and will require D-SNPs to integrate care coordination, member and clinician communications, and other activities in ways that go beyond the new federal requirements and the voluntary actions of D-SNPs studied here. The expectation is that these steps will build on the early indicators of success identified in this study and allow more dually eligible members the opportunity to receive health care in a single, streamlined program. Continued evaluation of these efforts is needed.

### Limitations

This study has limitations. Our analysis applies to Virginia; thus, our findings may not be generalizable to the US. In addition, potential bias due to survey nonresponse has been noted. Relatively small numbers of respondents may have reduced our ability to identify small and statistically significant differences across the groups. Although the health plans made the decision to adopt EAE and although we used weights defined from propensity score estimation to achieve balance across the 3 groups of respondents, we were unable to account for selection into Medicare plan type based on unobservable traits, which may have biased our estimates. Our survey was designed to assist Virginia Medicaid in evaluating its efforts toward alignment, and some of the questions on the survey specifically referenced the respondent’s Medicaid health plan. In D-SNPs with EAE, the member’s Medicare and Medicaid plans are the same from the member’s perspective.

## Conclusions

This cross-sectional study found that among dually enrolled Medicaid beneficiaries who used HCBS in Virginia, those in more highly integrated D-SNPs were significantly more likely to report satisfaction with some plan dimensions compared with beneficiaries in less integrated D-SNPs. There were no significant differences related to access to care. Our findings suggest that EAE is associated with reduced administrative complexity, as seen by increased customer service and overall plan satisfaction. However, our findings also suggest that attempts to increase Medicare-Medicaid integration through EAE will take more time, effort, or both to yield benefits for members’ access to care.

## Notes

Supplement 1. **eAppendix 1.** Survey Nonresponse **eTable 1.** Differences in Demographic Traits between Respondents and Nonrespondents, by Medicare Plan Type **eAppendix 2.** Additional Details on Methods **eFigure 1.** Distribution of propensity scores by treatment group (two treatments) **eFigure 2.** Distribution of propensity scores by treatment group (single treatment) **eAppendix 3.** Covariate Balance After Weighting **eTable 2.** Differences in Dually Eligible Beneficiaries’ Demographic Traits, ADLs, and Diagnosed Conditions, by Medicare Plan Type, Propensity Score Weighted Proportions and Means **eAppendix 4.** Sensitivity Analysis **eTable 3.** Dually Eligible Beneficiaries’ Access to Care, Overall, and by Type of Medicare Plan; Using Alternate Definitions **eTable 4.** Dually Eligible Beneficiaries’ Experience of Care, Overall, and by Type of Medicare Plan; Using Alternate Sample **eAppendix 5.** Survey Questions and Response Coding Used in Outcome Measure Construction **eTable 5.** Outcome Measure Construction **eAppendix 6.** Full Model Results **eTable 6.** Full model results of Dually Eligible Beneficiaries’ Experience of Care (for models reported in Table 2, D-SNP without EAE and D-SNP with EAE, relative to Traditional Medicare) **eTable 7.** Full Model Results of Dually Eligible Beneficiaries’ Out-of-Pocket Spending (for models reported in Table 3, D-SNP without and D-SNP with EAE, relative to Traditional Medicare) **eTable 8.** Full Model Results of Dually Eligible Beneficiaries’ Satisfaction (for models reported in Table 4, D-SNP without EAE and D-SNP with EAE, relative to Traditional Medicare) **eTable 9.** Full Model Results for Dually Eligible Beneficiaries’ Experience of Care (models reported in Table 2, Any D-SNP, relative to Traditional Medicare) **eTable 10.** Full Model Results of Dually Eligible Beneficiaries’ Out-of-Pocket Spending H(for models reported Table 3, Any D-SNP, relative to Traditional Medicare) **eTable 11.** Full Model Results of Dually Eligible Beneficiaries’ Satisfaction (from models reported Table 4, Any D-SNP, relative to Traditional Medicare)

Supplement 2. Data Sharing Statement
