# Supplementary material for: Beneficiary Experience of Care by Level of Integration in Dual Eligible Special Needs Plans
Source: JAMA Health Forum. 2024 Jun 7;5(6):e241383. doi: 10.1001/jamahealthforum.2024.1383 (PMC11161838; doi:10.1001/jamahealthforum.2024.1383)
Supplement: Supplement 1. — eAppendix 1. Survey Nonresponse eTable 1. Differences in Demographic Traits between Respondents and Nonrespondents, by Medicare Plan Type eAppendix 2. Additional Details on Methods eFigure 1. Distribution of propensity scores by treatment group (two treatments) eFigure 2. Distribution of propensity scores by treatment group (single treatment) eAppendix 3. Covariate Balance After Weighting eTable 2. Differences in Dually Eligible Beneficiaries’ Demographic Traits, ADLs, and Diagnosed Conditions, by Medicare Plan Type, Propensity Score Weighted Proportions and Means eAppendix 4. Sensitivity Analysis eTable 3. Dually Eligible Beneficiaries’ Access to Care, Overall, and by Type of Medicare Plan; Using Alternate Definitions eTable 4. Dually Eligible Beneficiaries’ Experience of Care, Overall, and by Type of Medicare Plan; Using Alternate Sample eAppendix 5. Survey Questions and Response Coding Used in Outcome Measure Construction eTable 5. Outcome Measure Construction eAppendix 6. Full Model Results eTable 6. Full model results of Dually Eligible Beneficiaries’ Experience of Care (for models reported in Table 2, D-SNP without EAE and D-SNP with EAE, relative to Traditional Medicare) eTable 7. Full Model Results of Dually Eligible Beneficiaries’ Out-of-Pocket Spending (for models reported in Table 3, D-SNP without and D-SNP with EAE, relative to Traditional Medicare) eTable 8. Full Model Results of Dually Eligible Beneficiaries’ Satisfaction (for models reported in Table 4, D-SNP without EAE and D-SNP with EAE, relative to Traditional Medicare) eTable 9. Full Model Results for Dually Eligible Beneficiaries’ Experience of Care (models reported in Table 2, Any D-SNP, relative to Traditional Medicare) eTable 10. Full Model Results of Dually Eligible Beneficiaries’ Out-of-Pocket Spending H(for models reported Table 3, Any D-SNP, relative to Traditional Medicare) eTable 11. Full Model Results of Dually Eligible Beneficiaries’ Satisfaction (from models reported Table 4, Any D-S [file jamahealthforum-e241383-s001.pdf]

Supplemental Online Content

Mellor JM, Cunningham PJ, Britton E, Behrens M, Urmi AF, Vega V. Beneficiary experience of care by level of integration in dual eligible special needs plans. *JAMA Health Forum.* Published online June 7, 2024. doi:10.1001/jamahealthforum.2024.1383

eAppendix 1. Survey Nonresponse

eTable 1. Differences in Demographic Traits between Respondents and Nonrespondents, by Medicare Plan Type

eAppendix 2. Additional Details on Methods

eFigure 1. Distribution of propensity scores by treatment group (two treatments)

eFigure 2. Distribution of propensity scores by treatment group (single treatment)

eAppendix 3. Covariate Balance After Weighting

eTable 2. Differences in Dually Eligible Beneficiaries' Demographic Traits, ADLs, and Diagnosed Conditions, by Medicare Plan Type, Propensity Score Weighted Proportions and Means

eAppendix 4. Sensitivity Analysis

eTable 3. Dually Eligible Beneficiaries' Access to Care, Overall, and by Type of Medicare Plan; Using Alternate Definitions

eTable 4. Dually Eligible Beneficiaries' Experience of Care, Overall, and by Type of Medicare Plan; Using Alternate Sample

eAppendix 5. Survey Questions and Response Coding Used in Outcome Measure Construction

eTable 5. Outcome Measure Construction

eAppendix 6. Full Model Results

eTable 6. Full model results of Dually Eligible Beneficiaries' Experience of Care (for models reported in Table 2, D-SNP without EAE and D-SNP with EAE, relative to Traditional Medicare)

eTable 7. Full Model Results of Dually Eligible Beneficiaries' Out-of-Pocket Spending (for models reported in Table 3, D-SNP without and D-SNP with EAE, relative to Traditional Medicare)

© 2024 Mellor JM et al. *JAMA Health Forum.*

eTable 8. Full Model Results of Dually Eligible Beneficiaries' Satisfaction (for models reported in Table 4, D-SNP without EAE and D-SNP with EAE, relative to Traditional Medicare)

eTable 9. Full Model Results for Dually Eligible Beneficiaries' Experience of Care (models reported in Table 2, Any D-SNP, relative to Traditional Medicare)

eTable 10. Full Model Results of Dually Eligible Beneficiaries' Out-of-Pocket Spending H(for models reported Table 3, Any D-SNP, relative to Traditional Medicare)

eTable 11. Full Model Results of Dually Eligible Beneficiaries' Satisfaction (from models reported Table 4, Any D-SNP, relative to Traditional Medicare)

This supplemental material has been provided by the authors to give readers additional information about their work.

© 2024 Mellor JM et al. *JAMA Health Forum.*

eAppendix 1. **Survey Nonresponse**

We mailed paper surveys to 2,713 dually enrolled members in traditional Medicare, 2,713 dually enrolled members in D-SNPs without EAE, and 1,774 in dually enrolled members in FIDE D-SNPS with EAE. The smaller number in the third group was due to the state having fewer FIDE D-SNP plans with EAE and fewer beneficiaries meeting the above inclusion criteria for the frame.

To assess for potential sample bias due to survey nonresponse, we used enrollment file data on age, sex, race, ethnicity, and residence to compare characteristics for the total starting sample with those of survey respondents for each survey stratum. Results are reported in eTable 1. For the traditional Medicare sample, there were a few marginally statistically significant differences between the respondent sample and the total sample. For the D-SNP with EAE sample, respondents were somewhat older, more likely to be White, and less likely to be Black compared with the total sample. For the D-SNP without EAE sample, respondents were also somewhat older, and more likely to be Hispanic and living in rural areas compared to the total sample. While these modest differences are unlikely to cause substantial bias in the results – especially when applying the statistical adjustments described below – nonresponse bias may result from other unknown differences between respondents and nonrespondents that are correlated with the outcome measures used in this analysis.

eTable 1. Differences in Demographic Traits between Respondents and Nonrespondents, by Medicare Plan Type[a]

	Traditional Medicare		D-SNPs with EAE[b]		D-SNPs without EAE[b]	
	Mailed survey	Responded to survey	Mailed survey	Responded to survey	Mailed survey	Responded to survey
Age %						
<45	8.38	8.20	7.85	4.63***	7.73	6.61
45-64	18.22	17.28	31.08	30.36	27.73	26.55
65-74	21.99	20.77	25.59	27.62	27.30	26.29
75+	51.41	53.75	35.49	37.39	37.24	40.55*
Age in years, no.	72.13	72.93	67.91	69.77***	68.57	69.98**
Female, %	70.54	73.47	67.83	68.78	70.30	71.99
Race, %[c]						
White	43.03	43.98	44.48	50.60**	37.12	40.16
Black	35.82	34.55	49.00	44.08**	53.48	50.59
Other race	21.15	21.47	6.52	5.32	9.40	9.25
Hispanic origin, %	1.59	2.79*	0.97	1.54	1.74	3.04**
Rural resident, %[d]	19.47	22.51*	27.64	30.53	24.13	29.06***

SOURCE: Authors' analyses of 2022 Commonwealth Coordinated Care Plus (CCCP) member survey data.

NOTES:

a) We tested for used to for differences in means for each trait or in the percent of respondents with each trait among survey respondents in each strata relative to all surveyed individuals (total sample), and use asterisks to indicate difference that were statistically significant as follows: *p < 0:10 **p < 0:05 ***p < 0:01.
b) EAE: Exclusively aligned enrollment.
c) Self-reported by respondents in Medicaid enrollment records.
d) Residence in a rural area based on RUCA codes developed by the USDA.

eAppendix 2. Additional Details on Methods

Below we provide additional details on the methods we used in the estimation of regression results reported in Tables 2, 3, and 4 of the main paper. Specifically, we combined multivariate regression analysis of outcomes with the use of propensity score weighting to compare dually eligible beneficiaries with different types of Medicare plans. Our work first compares three groups of members (two D-SNP treatments to traditional Medicare), and second compares two groups of members (one combined D-SNP treatment to traditional Medicare). We used a multi-step estimation described in (a) and (b) below to generate results reported in the manuscript and we checked that we obtained similar results using simultaneous estimation with generalized estimating equations, as described in (c) below.

(a) Comparing two different types of D-SNPs to traditional Medicare

A key study objective was to examine differences in outcomes by enrollment in two types of D-SNP (namely, D-SNPs with exclusively aligned enrollment and D-SNPs without exclusively aligned enrollment). To compare outcomes for members in either D-SNP coverage group to members in traditional Medicare and to those in the other D-SNP coverage group, we estimated the following regression equation:

(1) $Y_i = \beta_0 + \beta_1 X_i + \gamma \text{ D-SNP-EAE}_i + \delta \text{ D-SNP-OTHER}_I + \varepsilon_i$

where Y_i represents one of the outcomes we examine (pertaining to either patient experience of care, out-of-pocket spending, or patient satisfaction) for individual *i*, X_i is a set of respondent-level traits, and ε_i is a normally distributed error term. X_i includes variables measuring demographic traits, including six indicators for age (less than 45 years, 45-64 years, 70-74 years, 75-79 years, 80-84 years, and age 85 and older, relative to those age 65-70), an indicator for sex (female relative to male), two indicators for Black race and other race (relative to White race), Hispanic ethnicity (relative to non-Hispanic), two indicators for highest level of education (less than a high school education and high school education, relative to more than a high school education), three indicators for marital status (never married, separated/divorced, or widowed, relative to married), and an indicator for residence in a rural area (relative to non-rural). X_i also includes a count of limitations in up to six activities of daily living (ADLs) (including difficulties bathing/showering, dressing, eating, getting in and out of bed or chairs, walking, and toileting) and indicators for whether a physician or other health provider ever told the person that they had a heart condition, stroke, chronic obstructive pulmonary disease, cancer, diabetes, asthma, an intellectual or developmental disability, depression/anxiety or another mental health problem, a substance use disorder, dementia, or any other chronic disease. The key explanatory variables in Equation (1) are two indicators for members enrolled in D-SNPs with exclusively aligned enrollment (D-SNP-EAE_i) and members enrolled in D-SNPs without exclusively aligned enrollment (D-SNP-OTHER_I), relative to members in traditional Medicare.

In estimating Equation (1) we used propensity score weighting to account for non-random selection into types of Medicare coverage. For example, prior studies have found that, relative to those in traditional Medicare, those enrolled in D-SNPs are more likely to be people of color and less likely to reside in rural areas.[1-2] Given that our first set of analysis compares two treatment groups (D-SNP-EAE_i and D-SNP-OTHER_I) relative to the control group (traditional Medicare), we estimated propensity scores from a multinomial logistic regression model where the dependent variable indicated one of three categories of Medicare coverage.[3] As explanatory variables in the model we included the set of controls shown in X_i in Equation (1) and measuring demographic traits, clinical indicators, and the count of ADL limitations.

© 2024 Mellor JM et al. *JAMA Health Forum.*

eFigure 1. Distribution of propensity scores by treatment group (two treatment groups)

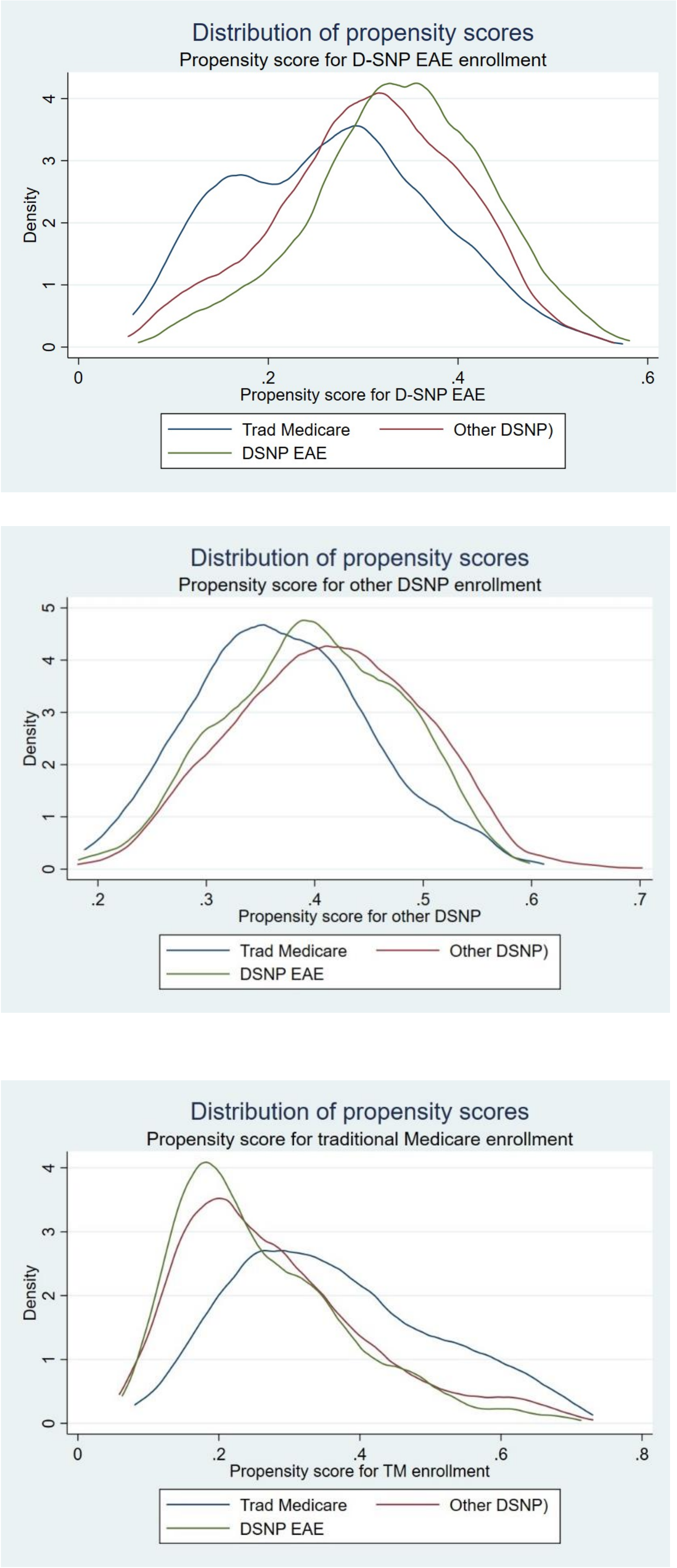

© 2024 Mellor JM et al. *JAMA Health Forum.*

We used the estimated model coefficients from the multinomial logistic regression to construct inverse probability of treatment weights (IPTW), as follows:

$$(2)\ w_i = \frac{1}{\Pr(M_i = j \mid X_i)}$$

where M_i is the Medicare coverage type for individual i and j takes values 1, 2, or 3 for traditional Medicare coverage, D-SNP coverage without EAE, and D-SNP coverage with EAE, respectively. Applying these weights in the estimation of Equation (1) thus places a higher weight on persons with lower probabilities of being in their observed category of Medicare coverage. We estimated robust standard errors of the coefficient estimates in the outcome (Equation 1) models.

We used the estimated coefficients from the IPTW weighted regression models of Equation (1) to report estimated differences in care by type of Medicare coverage in Tables 2, 3, and 4 of the paper, including the adjusted differences in outcomes between persons enrolled in D-SNPs with exclusively aligned enrollment relative to those with traditional Medicare (given by $\hat{\gamma}$) and adjusted differences between persons enrolled in D-SNPs without exclusively aligned enrollment relative to those with traditional Medicare (given by $\hat{\delta}$), and adjusted differences between persons in D-SNPs with exclusively aligned enrollment and those in other types of D-SNPs (given by $\hat{\gamma}$ - $\hat{\delta}$). For all estimates we report 95% confidence intervals and p-values.

(b) Comparing members in both types D-SNPs combined to traditional Medicare

We also examined differences in outcomes by enrollment in either type of D-SNP relative to members in traditional Medicare. We estimated the following regression equation:

$$(3)\quad Y_i = \beta_0 + \beta_1 X_i + \alpha\ \text{ANY-D-SNP}_i + \varepsilon_i$$

As in Equation (1) Y_i represents one of the outcomes we examine (pertaining to either patient experience of care, out-of-pocket spending, or patient satisfaction), X_i is the same set of respondent-level traits, and ε_i is a normally distributed error term. The key explanatory variable is ANY D-SNP, which equals one if the respondent is enrolled in either a D-SNP with EAE or another type of D-SNP and zero if the respondent is enrolled in traditional Medicare. As with the estimation of Equation (1), we also applied weights defined from a propensity score model. In this case, we estimated propensity scores from a logit regression of the single binary treatment (ANY-D-SNP) on Xi, and we calculated the propensity score (PS) as the probability of being in either type of D-SNP conditional on the person's characteristics (X).

We calculated inverse probability of treatment weights for each individual in either D-SNP plan as equal to 1/PS, and for each individual with traditional Medicare as equal to 1/(1−PS). This assigns larger weights to individuals in D-SNPs with a lower probability of being in a D-SNP as well as to individuals in traditional Medicare with a higher probability of being in a D-SNP. We estimated robust standard errors of the coefficient estimates in the outcome (Equation 3) models. Adjusted differences in outcomes between persons enrolled in either type of D-SNPs relative to those with traditional Medicare (given by $\hat{\alpha}$) are reported in the far-right column of Tables 2, 3, and 4.

© 2024 Mellor JM et al. *JAMA Health Forum.*

eFigure 2. Distribution of propensity scores by treatment group (single treatment group)

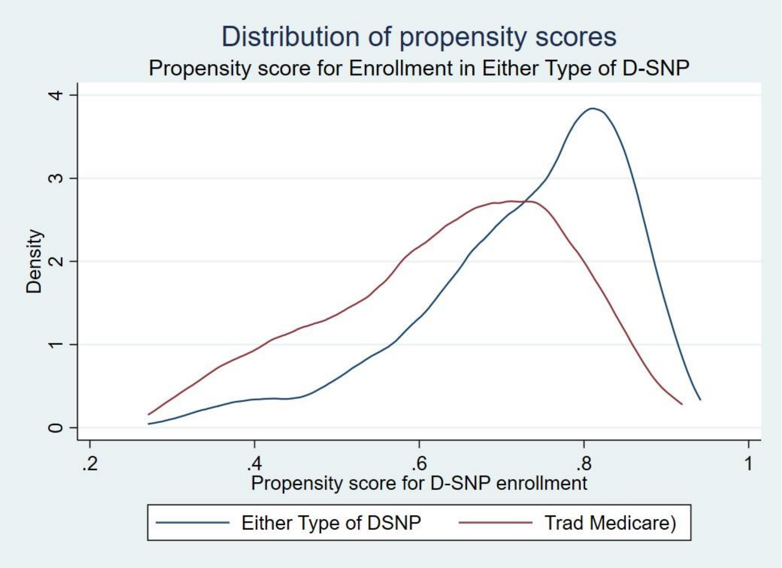

(c) Use of GMM Estimation

In addition to the multi-step approach described above, we also estimated all results from Tables 2, 3, and 4 using generalized method of moments (GMM) estimation that simultaneously estimate the propensity scores and outcome model, using the *teffects* procedure in Stata with IPT weighting and regression adjustment for both single and multivalued treatments. We obtained very similar results in support of our study conclusions (available on request).

References

1. Roberts ET, Mellor JM. Differences in care between Special Needs Plans and other Medicare coverage for dual eligibles. *Health Affairs*. 2022 Sep;41(9):1238–47.

2. Velasquez, DE, Orav, EJ, Figueroa, JF. Enrollment and Characteristics of Dual-Eligible Medicare and Medicaid Beneficiaries in Integrated Care Programs. *Health Affairs*. 2023 May; 42(5):683-692.

3. McCaffrey DF, Griffin BA, Almirall D, Slaughter ME, Ramchand R, Burgette LF. A tutorial on propensity score estimation for multiple treatments using generalized boosted models. *Statistics in Medicine*. 2013 Aug;32(19):3388-3414.

© 2024 Mellor JM et al. *JAMA Health Forum.*

eAppendix 3. Covariate Balance After Weighting

When observations were weighted by propensity score weights, we observed no statistically significant differences in member demographic traits, number of ADL limitations, and clinical diagnoses, as shown in eTable 2.

eTable 2. Differences in Dually Eligible Beneficiaries' Demographic Traits, ADLs, and Diagnosed Conditions, by Medicare Plan Type[a], Propensity Score Weighted Proportions and Means

	Traditional Medicare n=573	D-SNPs with EAE[b] n=583	p-value H_0: $Diff_{TM, EAE}=0$	D-SNPs without EAE n=757	p-value H_0: $Diff_{TM, OTH\_D-SNP}=0$
Age in years, number	70.55	70.59	0.97	70.92	0.69
Female, %	70.6	71.6	0.75	72.1	0.60
Race, %[c]					
Black	42.5	43.4	0.78	43.7	0.69
Other race	12.1	11.7	0.84	12.1	0.98
White (reference category)	45.4	44.9		44.2	
Hispanic origin, %[c]	2.5	2.9	0.72	2.5	0.99
Education, %					
More than high school (ref. category)	27.1	27.9		27	
High school	32.2	31.5	0.82	31.2	0.73
Less than high school	40.7	40.6	0.97	41.8	0.73
Marital status, %					
Married (ref. category)	16	16.4		16.2	
Never married	23.4	23.4	0.99	22.9	0.84
Separated or divorced	27.4	27.5	0.99	27.5	0.99
Widowed	33.2	32.7	0.87	33.4	0.95
Rural resident, %[d]	27.4	26.5	0.76	27.2	0.93
ADL limitations, number[e]	3.71	3.69	0.90	3.68	0.83
Diagnosed health conditions, %					
Heart condition	43.5	44.2	0.83	44.3	0.80
Stroke	25.2	25.8	0.83	24.2	0.70
COPD	23.4	25.4	0.49	25.1	0.54
Cancer	18.2	17.9	0.92	18.2	0.99
Diabetes	42.4	42.8	0.92	43.1	0.83
Asthma	26.7	27.2	0.85	27.3	0.80
Intellectual/Developmental Disability	18.3	16.6	0.48	17.9	0.85
Depression/anxiety	43.4	44.3	0.79	45	0.58
Dementia	18.6	17.9	0.77	18.6	0.99
Substance use disorder	3.7	3.2	0.69	3.6	0.96
Another chronic disease	17.4	17.2	0.95	17.9	0.83

SOURCE: Authors' analyses of 2022 Commonwealth Coordinated Care Plus (CCCP) member survey data.

NOTES:

a) P-values report results from tests of significant differences in the proportion of respondents with each trait (or variable mean in the case of the ADL limitation count) among dually eligible beneficiaries in each type of D-SNP relative to dually eligible beneficiaries enrolled in traditional Medicare. Estimates are based on 1,913 observations with complete data on the respondent characteristics in the far-left column.

© 2024 Mellor JM et al. *JAMA Health Forum.*

b) EAE: Exclusively aligned enrollment.
c) Self-reported by respondents. Other race respondents included persons who identified as Asian, Native American, more than one race, or some other race not indicated.
d) Residence in a rural area based on RUCA codes developed by the USDA.
e) Number of activities of daily living that a respondent reported difficulty performing alone and without using special equipment.

© 2024 Mellor JM et al. *JAMA Health Forum.*

eAppendix 4. Sensitivity Analysis

eTable 3. Dually Eligible Beneficiaries' Access to Care, Overall, and by Type of Medicare Plan; Using Alternate Definitions[a]

Outcome	Overall mean among dually eligible beneficiaries	Adjusted differences in outcomes in			
		D-SNPs with EAE[b] compared to traditional Medicare	D-SNPs without EAE compared to traditional Medicare	D-SNPs with EAE compared to D-SNPs without EAE	All D-SNPs compared to traditional Medicare
A lot of difficulty with:[c]					
Primary care	2.9	-2.1*	-0.3	-1.8	-1.1
Specialist care	4.2	1.1	1.8	-0.7	1.3
Mental health services	2.5	-0.2	0.4	-0.6	-0.1
Rx medications	2.6	0.4	-1.1	1.5	-0.6
Medical equipment and/or supplies	6.7	-1.7	0.4	-2.1	-0.8
Dental care	10.6	-2.4	-1.1	-1.3	-1.9
Vision care	4.9	-0.3	0.9	-1.2	0.1
Hearing care	4.0	-1.1	-1.2	0.1	-1.3
HCBS[d]	7.5	1.2	3.6**	-2.5	2.7**
Any type of care	25.6	-3.9	0.8	-4.7	-1.2
Number of types of care had some or a lot of difficulty[e]	1.207	-0.135	-0.120	-0.144	-0.143
Number of types of care had a lot of difficulty[c]	0.459	-0.061	0.038	-0.099	-0.018

SOURCE: Authors' analyses of 2022 Commonwealth Coordinated Care Plus (CCCP) member survey data.
NOTES:

a) Adjusted difference in the rate (or mean) of each outcome between the groups indicated in the column headings. Estimates were adjusted for the covariates in Table 1 and were by weighted propensity score weights. Adjusted differences are reported as percentage point differences, or differences in the number (bottom two rows). Significance levels based on robust standard errors.
b) EAE: Exclusively aligned enrollment.
c) Difficulty is defined as having a lot of difficulty, versus no difficulty/some difficulty/no need, accessing care.
d) HCBS (Home and Community Based Services), defined in the survey as "care and other in-home services and conveniences that help with daily activities (personal care services, adult day care, skilled nursing, etc.)
e) Difficulty is defined as having a lot of or some difficulty, versus no difficulty/ no need.

*p < 0:10 **p < 0:05 ***p < 0:01

© 2024 Mellor JM et al. *JAMA Health Forum.*

eTable 4. Dually Eligible Beneficiaries' Experience of Care, Overall, and by Type of Medicare Plan; Using Alternate Sample[a]

Outcome	Overall mean among dually eligible beneficiaries	Adjusted differences in outcomes in			
		D-SNPs with EAE[b] compared to traditional Medicare	D-SNPs without EAE compared to traditional Medicare	D-SNPs with EAE compared to D-SNPs without EAE	All D-SNPs compared to traditional Medicare
<u>Conditional on need, difficulty with:</u>[c]					
Primary care	3.3	-2.4*	-0.1	-2.3	-1.2
Specialist care	5.6	1.5	2.6*	-1.2	2.0
Mental health services	7.4	-1.5	0.9	-2.3	-0.6
Rx medications	2.9	0.4	-1.1	1.5	-0.6
Medical equipment and/or supplies	9.1	-2.4	1.0	-3.4	-0.9
Dental care	17.4	-5.7*	-3.3	-2.5	-4.7*
Vision care	7.4	-0.8	1.2	-2.1	-0.03
Hearing care	10.1	-3.4	-3.5	0.1	-2.9
HCBS[d]	9.2	1.5	4.5**	-3.0	3.3**

SOURCE: Authors' analyses of 2022 Commonwealth Coordinated Care Plus (CCCP) member survey data.

NOTES:
a) Adjusted difference in the rate of each outcome between the groups indicated in the column headings. Estimates were adjusted for the covariates in Table 1 and were by weighted propensity score weights. Adjusted differences are reported as percentage point differences. Significance levels based on robust standard errors.
b) EAE: Exclusively aligned enrollment.
c) Difficulty is defined as having some or a lot of difficulty, versus no difficulty, accessing care.
d) HCBS (Home and Community Based Services), defined in the survey as "care and other in-home services and conveniences that help with daily activities (personal care services, adult day care, skilled nursing, etc.)

*p < 0:10 **p < 0:05 ***p < 0:01

© 2024 Mellor JM et al. *JAMA Health Forum.*

eAppendix 5. Survey Questions and Response Coding Used in Outcome Measure Construction

For each outcome (dependent variable) used in the analysis reported in Tables 2-4, we report the text of the survey question and the details on the variable coding in eTable 3 below.

eTable 5. Outcome Measure Construction

Variable	Question	Coding
Difficulty with:	How much difficulty did you have getting:	
Primary care	Primary care	Binary, "A lot of difficulty" or "Some difficulty" versus "No difficulty" or "Did not need it"
Specialist care	Specialist care	" "
Mental health services	Mental health services	" "
Rx medications	Prescription medications	" "
Medical equipment and/or supplies	Medical equipment and/or supplies	" "
Dental care	Dental	" "
Vision care	Vision	" "
Hearing care	Hearing care	" "
HCBS[a]	Care and other in-home services and conveniences that help with daily activities (personal care services, adult day care, skilled nursing, etc.)	" "
Any type of care	Difficulty with any of the above	
Delays getting plan approval with:	In the last 6 months, how often did you experience delays in getting approval for services from your Medicaid health plan for the following:	
Medications	Medications	Binary: "Sometimes", "Usually", or "Always", versus "Never"
Specialist appts	Appointments with specialists	" "
HCBS	Care and other in-home services and conveniences that help with daily activities	" "
OOP Spending, $[b]	In the last 6 months, about how much did you spend out-of-pocket for your own healthcare?	Continuous ($)
Any OOP Spending, %	For which services did you pay out-of-pocket costs?	Check all that apply
Overall		Checked at least one
Physician Visits	Medical visit to a doctor	
Prescription drugs	Prescription drugs	
Dental care	Dental care	
Vision care	Vision care	

Variable	Question	Coding
Medical equipment	Medical equipment	
Feel OOP costs are a major financial burden, %	How much of a financial burden were the out-of-pocket costs you paid for your health care?	“Major burden” versus “Minor burden” and “Not a burden at all”
% Very satisfied with:		
Care Coordinator	Thinking about all the care coordination you may have received from you Medicaid health plan coordinator in the last 6 months…Overall are you currently satisfied or dissatisfied with the care coordination services you receive?	“Very Satisfied” versus “Somewhat Satisfied,” “Somewhat Dissatisfied,” “Very Dissatisfied”
PCP Choice	The choice of primary care physicians offered by your health plan?	“Very Satisfied” versus “Somewhat Satisfied,” “Somewhat Unsatisfied,” and “Very Unsatisfied”
Specialist choice	The choice of specialist physicians offered by your health plan?	“ “
Customer Service:		
Always gives needed info/help	How often did your health plan’s customer service give you the information or help you needed?	“Always” versus “Usually,” “Sometimes,” and “Never”
Always treats with courtesy/respect	How often did your health plan’s customer service treat you with courtesy and respect?	“ ”
Plan rating, on scale of 1-10	Using any number from 0-10, where 0 is the worst health plan possible and 10 is the best health plan possible, what number would you use to rate your Medicaid health plan?	“0- Worst health plan possible” through “10- Best health plan possible”
% rated plan a 10		“10- Best health plan possible”
% Strongly Agree:	Do you agree with the following statements?	
Know who to call about health/health care	I know who to call when I have questions about my health or healthcare	“Strongly Agree” versus “Agree,” “Disagree” and “Strongly Disagree”
Confident in understanding of health care system	I feel confident in my understanding of the healthcare system	“ “
Caring for health/chronic conditions is manageable	Caring for my health and chronic conditions is manageable	“ “

SOURCE: Author’s analyses of 2022 Commonwealth Coordinated Care Plus (CCCP) member survey data.
NOTES: a) HCBS (Home and Community Based Services), defined in the survey as “care and other in-home services and conveniences that help with daily activities (personal care services, adult day care, skilled nursing, etc.); b) OOP: Out-of-pocket.

© 2024 Mellor JM et al. *JAMA Health Forum.*

eAppendix 6. Full Regression Model Results

eTable 6: Full model results of Dually Eligible Beneficiaries' Experience of Care (for models reported in Table 2, D-SNP without EAE and D-SNP with EAE, relative to Traditional Medicare)

	Difficulty with[a]:				
	Primary care n=1885	Specialist care n=1885	Mental health services n=1883	RX medications n=1886	Medical equipment and/or supplies n=1884
Explanatory Variable					
D-SNP with EAE[b]	-0.0253	-0.00203	-0.00499	-0.0236	-0.0330
	(0.0194)	(0.0222)	(0.0145)	(0.0217)	(0.0246)
D-SNP without EAE	-0.0117	-0.0197	-0.00144	-0.0333*	-0.0312
	(0.0182)	(0.0194)	(0.0135)	(0.0195)	(0.0229)
Age in years					
<45	-0.0442	-0.0101	-0.0164	0.00453	0.0758
	(0.0398)	(0.0442)	(0.0345)	(0.0400)	(0.0500)
45-64	-0.0533*	-0.0288	-0.00230	0.0131	0.0318
	(0.0292)	(0.0307)	(0.0239)	(0.0287)	(0.0324)
70-74	-0.0422	-0.0660**	-0.0144	0.00547	0.0101
	(0.0338)	(0.0334)	(0.0257)	(0.0348)	(0.0364)
75-79	-0.0347	-0.0666*	-0.0354	0.0114	0.0513
	(0.0350)	(0.0348)	(0.0225)	(0.0358)	(0.0390)
80-84	-0.0446	-0.0268	-0.0508**	-0.00507	0.0360
	(0.0335)	(0.0366)	(0.0222)	(0.0351)	(0.0384)
85+	-0.0470	-0.0558	-0.0430*	-0.0278	-0.0182
	(0.0358)	(0.0365)	(0.0232)	(0.0341)	(0.0369)
Female	0.00806	-0.0142	0.00860	0.0140	-0.0250
	(0.0175)	(0.0198)	(0.0131)	(0.0199)	(0.0225)
Race					
Black	0.00546	-0.0337*	0.00517	-0.0219	-0.0367*
	(0.0168)	(0.0182)	(0.0128)	(0.0178)	(0.0210)
Other race	0.0115	0.0539	0.0578**	0.0103	-0.0438
	(0.0294)	(0.0356)	(0.0272)	(0.0323)	(0.0351)
Hispanic origin	0.0717	0.0278	-0.0208	0.0467	0.225***
	(0.0733)	(0.0769)	(0.0215)	(0.0807)	(0.0862)
Education					
Less than high school	-0.0189	-0.0502**	-0.0298*	-0.0297	-0.0816***
	(0.0203)	(0.0232)	(0.0154)	(0.0228)	(0.0253)
High school	-0.0176	-0.0493**	-0.0244	-0.0378*	-0.0477*
	(0.0207)	(0.0235)	(0.0172)	(0.0228)	(0.0264)
Marital status					
Never married	-0.00973	-0.0219	0.0243	-0.0297	-0.0277
	(0.0296)	(0.0329)	(0.0228)	(0.0333)	(0.0363)
Separated or divorced	-0.0151	0.00568	0.00176	0.00264	-0.00731
	(0.0255)	(0.0280)	(0.0184)	(0.0291)	(0.0331)
Widowed	-0.0249	-0.0131	-0.00697	-0.0154	0.00480
	(0.0252)	(0.0276)	(0.0167)	(0.0283)	(0.0322)

© 2024 Mellor JM et al. *JAMA Health Forum.*

	Difficulty with[a]:				
	Primary care n=1885	Specialist care n=1885	Mental health services n=1883	RX medications n=1886	Medical equipment and/or supplies n=1884
Rural resident	-0.0271*	-0.0432**	-0.00908	-0.0278	-0.0422**
	(0.0156)	(0.0171)	(0.0119)	(0.0177)	(0.0208)
ADL limitations	0.0145***	0.0109***	0.000202	0.0111***	0.0325***
	(0.00388)	(0.00421)	(0.00291)	(0.00419)	(0.00441)
Diagnosed health conditions					
Heart condition	0.0291*	0.00740	0.00457	0.0302*	0.0169
	(0.0159)	(0.0182)	(0.0122)	(0.0178)	(0.0202)
Stroke	-0.0255	-0.0143	-0.000781	-0.00921	-0.0161
	(0.0170)	(0.0203)	(0.0144)	(0.0202)	(0.0226)
COPD	-0.0177	-0.0431**	-0.0277*	-0.0463**	0.00765
	(0.0198)	(0.0216)	(0.0153)	(0.0205)	(0.0261)
Cancer	-0.00966	0.0175	0.0356**	0.0340	-0.0169
	(0.0196)	(0.0221)	(0.0176)	(0.0234)	(0.0244)
Diabetes	-0.00903	-0.0235	-0.00254	0.0347*	0.00646
	(0.0161)	(0.0179)	(0.0123)	(0.0180)	(0.0202)
Asthma	0.0202	0.0371	0.0152	0.0365*	0.0538**
	(0.0201)	(0.0232)	(0.0162)	(0.0218)	(0.0247)
Intellectual/Developmental Disability	-0.00261	0.000347	-0.000173	0.0251	0.0138
	(0.0221)	(0.0249)	(0.0176)	(0.0258)	(0.0288)
Depression/Anxiety	0.0498***	0.0688***	0.0834***	0.0541***	0.0342*
	(0.0171)	(0.0187)	(0.0132)	(0.0190)	(0.0205)
Dementia	-0.0501**	-0.0274	0.00452	-0.0260	-0.0593**
	(0.0206)	(0.0250)	(0.0150)	(0.0226)	(0.0261)
Substance use disorder	0.0663	0.0958	0.0459	0.0770	0.0403
	(0.0534)	(0.0588)	(0.0505)	(0.0579)	(0.0613)
Another chronic disease	-0.00973	0.0471*	-0.00573	-0.0160	0.0606**
	(0.0208)	(0.0252)	(0.0160)	(0.0217)	(0.0274)
Constant	0.114***	0.186***	0.0402	0.0906*	0.133**
	(0.0419)	(0.0483)	(0.0316)	(0.0462)	(0.0525)

NOTES:

a) Difficulty is defined as having a lot of difficulty or some difficulty, versus no difficulty/no need.

b) EAE: Exclusively aligned enrollment.

*p < 0:10 **p < 0:05 ***p < 0:01

© 2024 Mellor JM et al. *JAMA Health Forum.*

eTable 6 continued

	Difficulty with[a]:				
	Dental care n=1885	Vision care n=1885	Hearing care n=1880	HCBS[b] n=1886	Any type of care n=1882
Explanatory variable					
D-SNP without EAE[c]	-0.0112	-0.0103	-0.0248	0.0231	-0.0119
	(0.0228)	(0.0192)	(0.0157)	(0.0219)	(0.0283)
D-SNP with EAE	-0.0145	-0.0102	-0.00755	-0.00298	-0.0530*
	(0.0250)	(0.0213)	(0.0180)	(0.0234)	(0.0304)
Age in years					
<45	0.158***	0.0345	0.00366	-0.0807*	0.0777
	(0.0562)	(0.0386)	(0.0312)	(0.0444)	(0.0621)
45-64	0.0574*	0.0570**	0.00296	-0.0663**	-0.00508
	(0.0342)	(0.0257)	(0.0209)	(0.0322)	(0.0410)
70-74	0.0184	0.0803**	0.0147	-0.0434	-0.0409
	(0.0373)	(0.0312)	(0.0245)	(0.0374)	(0.0461)
75-79	-0.0205	0.0187	0.0334	-0.0215	0.0183
	(0.0370)	(0.0280)	(0.0256)	(0.0387)	(0.0490)
80-84	0.0224	0.0617**	0.0668**	0.0371	0.0141
	(0.0390)	(0.0308)	(0.0289)	(0.0410)	(0.0492)
85+	-0.00494	0.0698**	0.0775**	0.0115	0.0223
	(0.0385)	(0.0327)	(0.0304)	(0.0405)	(0.0485)
Female	0.0285	0.000651	0.0131	-0.00558	-0.0145
	(0.0220)	(0.0186)	(0.0155)	(0.0219)	(0.0281)
Race					
Black	0.00674	0.000976	-0.0145	-0.0384**	-0.00199
	(0.0215)	(0.0174)	(0.0150)	(0.0194)	(0.0262)
Other race	0.0685*	0.0813**	0.0678**	-0.0227	0.0312
	(0.0386)	(0.0363)	(0.0329)	(0.0376)	(0.0453)
Hispanic origin	0.0431	0.0341	-0.0128	0.0870	0.117
	(0.0766)	(0.0740)	(0.0670)	(0.0772)	(0.0862)
Education					
Less than high school	-0.0383	-0.0257	-0.0139	-0.0897***	-0.142***
	(0.0264)	(0.0219)	(0.0185)	(0.0251)	(0.0319)
High school	-0.0382	-0.0235	0.00334	-0.0564**	-0.0930***
	(0.0262)	(0.0220)	(0.0186)	(0.0255)	(0.0317)
Marital status					
Never married	0.00510	-0.0237	-0.00315	0.00122	-0.0221
	(0.0354)	(0.0313)	(0.0257)	(0.0364)	(0.0437)
Separated or divorced	0.0192	0.00615	0.00775	-0.0317	0.00845
	(0.0307)	(0.0274)	(0.0213)	(0.0319)	(0.0390)
Widowed	0.00346	0.00329	-0.00835	-0.0215	0.00843
	(0.0302)	(0.0268)	(0.0233)	(0.0330)	(0.0399)
Rural resident	-0.0393*	-0.0477***	-0.0246*	-0.0315	-0.0969***
	(0.0207)	(0.0161)	(0.0138)	(0.0198)	(0.0269)
ADL limitations	0.00622	0.00465	0.00794**	0.0212***	0.0259***
	(0.00503)	(0.00417)	(0.00337)	(0.00448)	(0.00610)

© 2024 Mellor JM et al. *JAMA Health Forum.*

	Difficulty with[a]:				
	Dental care n=1885	Vision care n=1885	Hearing care n=1880	HCBS[b] n=1886	Any type of care n=1882
Diagnosed health conditions					
Heart condition	0.0336	0.0127	0.0180	-0.0157	0.0412
	(0.0208)	(0.0175)	(0.0151)	(0.0197)	(0.0255)
Stroke	-0.0153	0.0124	0.00525	-0.000518	-0.0171
	(0.0228)	(0.0210)	(0.0182)	(0.0225)	(0.0281)
COPD	-0.0436*	-0.0107	-0.0278	-0.0179	0.000841
	(0.0239)	(0.0230)	(0.0170)	(0.0246)	(0.0310)
Cancer	-0.00853	0.0197	0.0185	-0.0223	0.0212
	(0.0246)	(0.0220)	(0.0201)	(0.0232)	(0.0309)
Diabetes	-0.00113	-0.0169	0.0130	-0.0179	0.0235
	(0.0208)	(0.0176)	(0.0149)	(0.0193)	(0.0249)
Asthma	0.0713***	0.0585**	0.0252	0.0399*	0.0677**
	(0.0260)	(0.0230)	(0.0196)	(0.0242)	(0.0294)
Intellectual/Developmental Disability	0.0415	0.0531**	0.0503**	0.0451	0.0857**
	(0.0299)	(0.0265)	(0.0236)	(0.0295)	(0.0341)
Depression/Anxiety	0.0969***	0.0765***	0.0550***	0.0564***	0.133***
	(0.0218)	(0.0182)	(0.0154)	(0.0203)	(0.0265)
Dementia	-0.0507*	-0.0809***	-0.0589***	-0.00761	-0.0465
	(0.0261)	(0.0218)	(0.0202)	(0.0276)	(0.0333)
Substance use disorder	0.0654	0.0497	0.0560	0.138**	0.133**
	(0.0632)	(0.0571)	(0.0521)	(0.0687)	(0.0625)
Another chronic disease	0.0395	0.000759	-0.00981	0.00711	0.0633**
	(0.0281)	(0.0229)	(0.0185)	(0.0246)	(0.0318)
Constant	0.0902*	0.0388	0.00134	0.183***	0.368***
	(0.0518)	(0.0444)	(0.0343)	(0.0505)	(0.0636)

NOTES:

a) Difficulty is defined as having a lot of difficulty or some difficulty, versus no difficulty/no need.

b) HCBS (Home and Community Based Services), defined in the survey as "care and other in-home services and conveniences that help with daily activities (personal care services, adult day care, skilled nursing, etc.)

c) EAE: Exclusively aligned enrollment.

*p < 0:10 **p < 0:05 ***p < 0:01

© 2024 Mellor JM et al. *JAMA Health Forum.*

eTable 6 continued

	Delays getting plan approvals: For medications n=1844	Delays getting plan approvals: For specialist appts n=1841	Delays getting plan approvals: For HCBS[b] n=1846
Explanatory Variable			
D-SNP without EAE[c]	0.00790	0.0501[*]	0.0231
	(0.0274)	(0.0262)	(0.0282)
D-SNP with EAE	-0.0160	0.0170	0.00614
	(0.0294)	(0.0281)	(0.0297)
Age in years			
<45	-0.0547	-0.0476	-0.123[**]
	(0.0574)	(0.0583)	(0.0574)
45-64	0.0380	-0.0312	-0.0856[**]
	(0.0405)	(0.0393)	(0.0413)
70-74	0.0247	-0.0326	-0.0184
	(0.0457)	(0.0442)	(0.0476)
75-79	-0.00470	-0.0716	-0.0151
	(0.0476)	(0.0456)	(0.0500)
80-84	-0.0216	-0.0357	0.0476
	(0.0451)	(0.0442)	(0.0483)
85+	0.0115	-0.0332	-0.0170
	(0.0457)	(0.0452)	(0.0479)
Female	0.0511[*]	0.0305	0.0351
	(0.0270)	(0.0258)	(0.0276)
Race			
Black	0.00992	0.000541	0.00775
	(0.0254)	(0.0241)	(0.0256)
Other race	0.0603	0.132[***]	-0.00345
	(0.0430)	(0.0433)	(0.0419)
Hispanic origin	0.0676	0.129	0.0637
	(0.0780)	(0.0821)	(0.0809)
Education			
Less than high school	-0.0308	-0.0517[*]	-0.0535[*]
	(0.0303)	(0.0294)	(0.0306)
High school	-0.0682[**]	-0.0567[*]	-0.0352
	(0.0308)	(0.0299)	(0.0314)
Marital status			
Never married	-0.0301	-0.0290	-0.0751[*]
	(0.0410)	(0.0389)	(0.0426)
Separated or divorced	0.0222	0.00266	-0.0425
	(0.0376)	(0.0351)	(0.0390)
Widowed	-0.0404	-0.00914	-0.0677[*]
	(0.0369)	(0.0353)	(0.0390)
Rural resident	0.0278	0.00794	-0.0137

© 2024 Mellor JM et al. *JAMA Health Forum.*

	Delays getting plan approvals: For medications n=1844	Delays getting plan approvals: For specialist appts n=1841	Delays getting plan approvals: For HCBS[b] n=1846
	(0.0264)	(0.0250)	(0.0270)
ADL limitations	0.0166***	0.00387	0.0177***
	(0.00578)	(0.00576)	(0.00601)
Diagnosed health conditions			
Heart condition	0.00781	0.0511**	0.0189
	(0.0242)	(0.0234)	(0.0247)
Stroke	0.00314	0.00383	-0.0299
	(0.0274)	(0.0261)	(0.0272)
COPD	-0.0256	-0.0471*	-0.0318
	(0.0301)	(0.0285)	(0.0303)
Cancer	0.0263	0.0154	-0.00611
	(0.0299)	(0.0285)	(0.0298)
Diabetes	0.0897***	0.0296	0.0613**
	(0.0240)	(0.0230)	(0.0244)
Asthma	0.112***	0.0944***	0.0798***
	(0.0294)	(0.0285)	(0.0291)
Intellectual/Developmental Disability	0.0971***	0.0828**	0.0968***
	(0.0336)	(0.0324)	(0.0329)
Depression/Anxiety	0.0480*	0.0194	0.00743
	(0.0250)	(0.0236)	(0.0251)
Dementia	-0.0656**	-0.0546*	-0.0643**
	(0.0307)	(0.0303)	(0.0324)
Substance use disorder	-0.0408	0.0234	0.0542
	(0.0635)	(0.0678)	(0.0705)
Another chronic disease	0.0326	-0.0513*	-0.0342
	(0.0301)	(0.0284)	(0.0303)
Constant	0.132**	0.211***	0.292***
	(0.0593)	(0.0580)	(0.0617)

NOTES:
a) Difficulty is defined as having a lot of difficulty or some difficulty, versus no difficulty/no need.
b) HCBS (Home and Community Based Services), defined in the survey as "care and other in-home services and conveniences that help with daily activities (personal care services, adult day care, skilled nursing, etc.)
c) EAE: Exclusively aligned enrollment.
*p < 0:10 **p < 0:05 ***p < 0:01

© 2024 Mellor JM et al. *JAMA Health Forum.*

eTable 7: Full Model Results of Dually Eligible Beneficiaries' Out-of-Pocket Spending (for models reported in Table 3, D-SNP without and D-SNP with EAE, relative to Traditional Medicare)

	Out-of-Pocket Spending				
	log (OOP[a] Spending) n=1694	Overall n=1821	Physician Visits n=1913	Prescription drugs n=1913	Dental care n=1913
Explanatory Variable					
D-SNP without EAE[b]	-0.820***	-0.114***	0.0145	-0.0443**	-0.0455***
	(0.173)	(0.0294)	(0.0169)	(0.0222)	(0.0172)
D-SNP with EAE	-0.786***	-0.117***	0.0143	-0.0623***	-0.0684***
	(0.187)	(0.0321)	(0.0181)	(0.0229)	(0.0175)
Age in years					
<45	0.250	0.00215	0.0459	-0.0251	0.0455
	(0.368)	(0.0648)	(0.0318)	(0.0445)	(0.0392)
45-64	-0.0474	-0.0493	0.0316	-0.0328	-0.00612
	(0.243)	(0.0428)	(0.0217)	(0.0336)	(0.0252)
70-74	-0.113	-0.0539	0.00860	-0.0430	-0.00269
	(0.285)	(0.0494)	(0.0278)	(0.0358)	(0.0270)
75-79	-0.179	-0.0429	-0.0246	-0.0596	-0.0575**
	(0.281)	(0.0519)	(0.0218)	(0.0367)	(0.0237)
80-84	0.00515	-0.0105	-0.0112	-0.0657*	-0.0110
	(0.286)	(0.0503)	(0.0238)	(0.0350)	(0.0267)
85+	0.371	0.0753	0.0269	-0.0173	-0.0127
	(0.282)	(0.0502)	(0.0257)	(0.0361)	(0.0254)
Female	0.0126	0.00613	-0.0208	-0.00464	0.00809
	(0.167)	(0.0292)	(0.0179)	(0.0214)	(0.0155)
Race					
Black	-0.391**	-0.0536*	0.0172	-0.00606	-0.0387**
	(0.159)	(0.0275)	(0.0148)	(0.0204)	(0.0154)
Other race	0.307	0.0515	0.0148	-0.00334	0.0255
	(0.287)	(0.0475)	(0.0281)	(0.0294)	(0.0263)
Hispanic origin	-0.0202	0.112	0.0152	0.105	-0.000484
	(0.423)	(0.0783)	(0.0510)	(0.0800)	(0.0350)
Education					
Less than high school	-0.668***	-0.112***	-0.0171	-0.0188	-0.0508***
	(0.186)	(0.0322)	(0.0182)	(0.0230)	(0.0178)
High school	-0.382**	-0.0560*	-0.0395**	0.00470	-0.0466**
	(0.194)	(0.0330)	(0.0182)	(0.0253)	(0.0193)
Marital status					
Never married	-0.109	-0.0268	-0.00547	-0.0304	-0.00514
	(0.256)	(0.0450)	(0.0249)	(0.0319)	(0.0245)
Separated or divorced	-0.146	-0.0231	0.00312	-0.0318	0.0105
	(0.241)	(0.0406)	(0.0222)	(0.0303)	(0.0235)
Widowed	0.0244	-0.0210	0.0278	-0.0257	0.00401
	(0.239)	(0.0414)	(0.0198)	(0.0287)	(0.0214)
Rural resident	-0.133	-0.00186	0.0217	0.0111	0.00860
	(0.158)	(0.0284)	(0.0167)	(0.0210)	(0.0155)
ADL limitations	0.178***	0.0261***	-0.00197	0.00756*	-0.00161

© 2024 Mellor JM et al. *JAMA Health Forum.*

	Out-of-Pocket Spending				
	log (OOP[a] Spending)	Overall	Physician Visits	Prescription drugs	Dental care
	n=1694	n=1821	n=1913	n=1913	n=1913
	(0.0366)	(0.00635)	(0.00364)	(0.00446)	(0.00371)
Diagnosed health conditions					
Heart condition	0.294*	0.0617**	0.0273*	0.0170	0.000925
	(0.151)	(0.0264)	(0.0153)	(0.0188)	(0.0135)
Stroke	0.0851	0.00654	-0.0182	0.0304	0.0148
	(0.170)	(0.0294)	(0.0168)	(0.0225)	(0.0172)
COPD	-0.381**	-0.0635**	-0.0263	-0.00839	-0.0551***
	(0.175)	(0.0317)	(0.0169)	(0.0228)	(0.0145)
Cancer	-0.0263	-0.0151	0.0136	-0.0202	0.00747
	(0.189)	(0.0321)	(0.0187)	(0.0219)	(0.0182)
Diabetes	-0.0919	-0.00709	-0.0203	0.00972	-0.0116
	(0.148)	(0.0257)	(0.0145)	(0.0186)	(0.0141)
Asthma	0.159	0.0320	0.0290	0.0193	0.0381**
	(0.170)	(0.0298)	(0.0182)	(0.0220)	(0.0165)
Intellectual/Developmental Disability	-0.00879	0.0111	-0.00530	-0.0490**	0.0211
	(0.197)	(0.0349)	(0.0181)	(0.0220)	(0.0195)
Depression/Anxiety	0.324**	0.0596**	0.00894	0.0363*	0.0108
	(0.157)	(0.0269)	(0.0148)	(0.0195)	(0.0146)
Dementia	-0.0274	-0.0266	0.00323	-0.0177	-0.0226
	(0.198)	(0.0346)	(0.0187)	(0.0232)	(0.0158)
Substance use disorder	0.0390	0.0246	0.0158	0.0207	0.0277
	(0.409)	(0.0694)	(0.0405)	(0.0497)	(0.0393)
Another chronic disease	0.699***	0.103***	0.0230	0.0141	0.00688
	(0.207)	(0.0326)	(0.0209)	(0.0242)	(0.0185)
Constant	2.338***	0.484***	0.0672**	0.191***	0.165***
	(0.371)	(0.0646)	(0.0339)	(0.0476)	(0.0377)

NOTES:

a) OOP: out-of-pocket

b) EAE: Exclusively aligned enrollment.

*p < 0:10 **p < 0:05 ***p < 0:01

© 2024 Mellor JM et al. *JAMA Health Forum.*

eTable 7 continued

	Out-of-Pocket Spending		
	Vision care n=1913	Medical equipment n=1913	Feel OOP[a] costs are a major financial burden n=1794
Explanatory Variable			
D-SNP without EAE[b]	-0.00745	-0.0327*	-0.0373*
	(0.0163)	(0.0176)	(0.0219)
D-SNP EAE	-0.0360**	-0.0450**	-0.0635***
	(0.0155)	(0.0184)	(0.0230)
Age in years			
<45	-0.00619	0.0113	-0.0817*
	(0.0272)	(0.0362)	(0.0430)
45-64	0.0171	-0.0122	-0.0179
	(0.0198)	(0.0252)	(0.0334)
70-74	0.0525*	-0.0544**	-0.0565
	(0.0282)	(0.0272)	(0.0362)
75-79	-0.00160	-0.0247	-0.0570
	(0.0202)	(0.0308)	(0.0378)
80-84	-0.00431	-0.0507*	-0.0596
	(0.0210)	(0.0291)	(0.0383)
85+	0.0429*	-0.0265	-0.0798**
	(0.0246)	(0.0315)	(0.0365)
Female	0.0154	0.00797	0.00227
	(0.0153)	(0.0162)	(0.0207)
Race			
Black	0.000571	-0.0540***	-0.0233
	(0.0138)	(0.0153)	(0.0189)
Other race	0.00749	-0.0277	0.0270
	(0.0206)	(0.0284)	(0.0375)
Hispanic origin	-0.0275	0.0205	-0.0238
	(0.0303)	(0.0518)	(0.0526)
Education			
Less than high school	0.0102	-0.0316*	-0.0828***
	(0.0153)	(0.0182)	(0.0242)
High school	0.0161	-0.00496	-0.0494*
	(0.0163)	(0.0200)	(0.0253)
Marital status			
Never married	-0.0213	-0.0363	0.00915
	(0.0206)	(0.0250)	(0.0330)
Separated or divorced	-0.0122	0.00206	0.00996
	(0.0204)	(0.0248)	(0.0293)
Widowed	-0.0114	0.00303	0.0278
	(0.0195)	(0.0234)	(0.0282)
Rural resident	0.00432	-0.0311**	-0.0663***
	(0.0147)	(0.0153)	(0.0176)

© 2024 Mellor JM et al. *JAMA Health Forum.*

	Out-of-Pocket Spending		
	Vision care n=1913	Medical equipment n=1913	Feel OOP[a] costs are a major financial burden n=1794
ADL limitations	0.000397	0.0166***	0.0179***
	(0.00341)	(0.00323)	(0.00412)
Diagnosed health conditions			
Heart condition	0.0229*	0.0377**	0.0297
	(0.0127)	(0.0161)	(0.0182)
Stroke	-0.0189	-0.00224	0.0170
	(0.0134)	(0.0172)	(0.0228)
COPD	-0.0136	-0.0304	-0.0220
	(0.0149)	(0.0205)	(0.0231)
Cancer	-0.00968	0.00122	0.0341
	(0.0148)	(0.0187)	(0.0242)
Diabetes	0.00298	0.000782	-0.0205
	(0.0126)	(0.0149)	(0.0187)
Asthma	0.0229	-0.0122	0.0551**
	(0.0155)	(0.0181)	(0.0234)
Intellectual/Developmental Disability	0.0131	0.0186	-0.00588
	(0.0170)	(0.0216)	(0.0252)
Depression/Anxiety	0.0111	0.00672	0.0343*
	(0.0126)	(0.0152)	(0.0200)
Dementia	-0.0275*	-0.0115	-0.0156
	(0.0155)	(0.0203)	(0.0247)
Substance use disorder	-0.0235	0.0240	0.0328
	(0.0250)	(0.0522)	(0.0577)
Another chronic disease	0.0173	0.0865***	0.0678**
	(0.0197)	(0.0234)	(0.0272)
Constant	0.0406	0.107***	0.176***
	(0.0279)	(0.0374)	(0.0496)

NOTES:

a) OOP: out-of-pocket

b) EAE: Exclusively aligned enrollment.

*p < 0:10 **p < 0:05 ***p < 0:01

© 2024 Mellor JM et al. *JAMA Health Forum.*

eTable 8: Full Model Results of Dually Eligible Beneficiaries' Satisfaction (for models reported in Table 4, D-SNP without EAE and D-SNP with EAE, relative to Traditional Medicare)

	Very satisfied with:				
	Care Coordinator n=1708	PCP choice n=1871	Specialist choice n=1839	Customer service: Always gives needed info/help n=1080	Customer service: Always treats with courtesy/ respect n=1078
Explanatory Variables					
D-SNP without EAE[a]	0.0119	-0.000672	-0.0349	0.0131	0.00109
	(0.0286)	(0.0293)	(0.0297)	(0.0399)	(0.0330)
D-SNP with EAE	-0.0215	0.0147	0.00222	0.0449	0.0688**
	(0.0311)	(0.0318)	(0.0324)	(0.0408)	(0.0322)
Age in years					
<45	-0.0707	-0.117*	-0.0667	-0.116	0.0330
	(0.0664)	(0.0671)	(0.0677)	(0.0837)	(0.0706)
45-64	-0.00940	-0.00308	0.0165	0.103*	0.0546
	(0.0400)	(0.0414)	(0.0426)	(0.0544)	(0.0388)
70-74	-0.0391	-0.00632	0.00836	-0.00365	-0.0344
	(0.0454)	(0.0470)	(0.0480)	(0.0615)	(0.0507)
75-79	-0.0443	-0.0962*	-0.0763	0.0277	0.0128
	(0.0472)	(0.0515)	(0.0522)	(0.0680)	(0.0558)
80-84	-0.113**	0.0202	0.0267	-0.0321	-0.0415
	(0.0485)	(0.0481)	(0.0489)	(0.0642)	(0.0522)
85+	-0.0865*	-0.0713	-0.0880*	-0.0128	-0.0511
	(0.0470)	(0.0494)	(0.0506)	(0.0651)	(0.0514)
Female	0.0298	0.0330	0.0353	0.0358	0.0207
	(0.0295)	(0.0294)	(0.0297)	(0.0379)	(0.0317)
Race					
Black	0.0420	0.0556**	0.0384	0.0372	0.0163
	(0.0265)	(0.0269)	(0.0274)	(0.0363)	(0.0267)
Other race	-0.0760	-0.0173	-0.0481	-0.209***	-0.260***
	(0.0488)	(0.0482)	(0.0484)	(0.0567)	(0.0570)
Hispanic origin	-0.0233	0.000634	-0.0200	-0.0510	-0.0436
	(0.0834)	(0.0858)	(0.0844)	(0.101)	(0.0931)
Education					
Less than high school	0.140***	0.00141	0.0269	0.0478	0.0786**
	(0.0324)	(0.0328)	(0.0333)	(0.0411)	(0.0357)
High school	0.0796**	0.0572*	0.0678**	0.0639	0.0444
	(0.0333)	(0.0319)	(0.0324)	(0.0423)	(0.0338)
Marital status					
Never married	-0.0542	-0.0895**	-0.0863*	0.00835	-0.0119
	(0.0436)	(0.0442)	(0.0447)	(0.0577)	(0.0474)
Separated or divorced	-0.00997	-0.0714*	-0.0816**	0.0744	-0.0243
	(0.0395)	(0.0399)	(0.0407)	(0.0531)	(0.0409)
Widowed	-0.00104	-0.0376	-0.0502	0.0509	-0.0405

© 2024 Mellor JM et al. *JAMA Health Forum.*

	Very satisfied with:				
	Care Coordinator n=1708	PCP choice n=1871	Specialist choice n=1839	Customer service: Always gives needed info/help n=1080	Customer service: Always treats with courtesy/ respect n=1078
	(0.0409)	(0.0401)	(0.0407)	(0.0535)	(0.0438)
Rural resident	0.0736***	0.0177	0.00810	0.0438	0.00203
	(0.0264)	(0.0275)	(0.0282)	(0.0383)	(0.0281)
ADL limitations	-0.0113*	-0.0155**	-0.0117*	0.00835	-0.00225
	(0.00630)	(0.00636)	(0.00648)	(0.00849)	(0.00641)
Diagnosed health conditions					
Heart condition	0.0430*	0.00912	0.0255	-0.00186	-0.0118
	(0.0260)	(0.0259)	(0.0264)	(0.0341)	(0.0270)
Stroke	-0.0318	-0.0366	-0.00153	0.00579	-0.000243
	(0.0293)	(0.0296)	(0.0301)	(0.0402)	(0.0303)
COPD	0.00121	0.0629**	0.0594*	0.00748	-0.00744
	(0.0308)	(0.0303)	(0.0312)	(0.0405)	(0.0325)
Cancer	-0.0139	-0.0195	-0.0272	0.00738	-0.0571
	(0.0314)	(0.0314)	(0.0319)	(0.0425)	(0.0352)
Diabetes	-0.0594**	-0.0102	-0.00340	-0.0714**	-0.0180
	(0.0252)	(0.0254)	(0.0260)	(0.0330)	(0.0262)
Asthma	0.0222	0.0142	0.0120	-0.0293	0.0110
	(0.0296)	(0.0291)	(0.0298)	(0.0370)	(0.0300)
Intellectual/Developmental Disability	-0.0348	0.0490	0.00766	0.0966**	-0.0407
	(0.0346)	(0.0355)	(0.0362)	(0.0443)	(0.0368)
Depression/Anxiety	-0.0714***	-0.0721***	-0.0747***	-0.0607*	0.0258
	(0.0260)	(0.0267)	(0.0270)	(0.0354)	(0.0275)
Dementia	0.0368	0.00484	0.0318	-0.0445	-0.0412
	(0.0336)	(0.0347)	(0.0354)	(0.0451)	(0.0394)
Substance use disorder	-0.142*	-0.189***	-0.202***	-0.139*	-0.166**
	(0.0784)	(0.0679)	(0.0677)	(0.0810)	(0.0804)
Another chronic disease	-0.0448	-0.00981	-0.0474	-0.0386	-0.00832
	(0.0328)	(0.0329)	(0.0333)	(0.0428)	(0.0340)
Constant	0.700***	0.692***	0.651***	0.340***	0.790***
	(0.0629)	(0.0637)	(0.0647)	(0.0882)	(0.0677)

NOTES: a) EAE: Exclusively aligned enrollment. *p < 0:10 **p < 0:05 ***p < 0:01

eTable 8 continued

	Plan rating, on scale of 1-10 n=1884	Rated plan a 10 n=1884	Strongly agree: Know who to call about health/ health care choice n=1894	Strongly agree: Confident in understanding of health care system n=1893	Strongly agree: Caring for health/ chronic condition is manageable n=1892
Explanatory Variable					
D-SNP without EAE[a]	0.116	0.0380	-0.00994	0.0279	0.0256
	(0.103)	(0.0288)	(0.0295)	(0.0276)	(0.0276)
D-SNP EAE	0.295***	0.0854***	0.0484	0.0538*	0.0529*
	(0.108)	(0.0308)	(0.0316)	(0.0294)	(0.0296)
Age in years					
<45	-0.623***	-0.163***	0.0101	0.0256	0.0310
	(0.216)	(0.0600)	(0.0648)	(0.0601)	(0.0598)
45-64	-0.0928	-0.0218	0.0446	0.0787*	0.0343
	(0.152)	(0.0419)	(0.0425)	(0.0406)	(0.0397)
70-74	-0.0653	0.0127	-0.0222	0.0536	0.0435
	(0.158)	(0.0470)	(0.0485)	(0.0464)	(0.0460)
75-79	-0.0598	-0.0309	-0.0473	-0.0261	-0.0432
	(0.167)	(0.0510)	(0.0515)	(0.0476)	(0.0481)
80-84	-0.340**	-0.0942*	-0.0455	-0.0179	-0.0138
	(0.173)	(0.0490)	(0.0502)	(0.0468)	(0.0470)
85+	-0.202	-0.131***	-0.111**	-0.0427	-0.0628
	(0.164)	(0.0481)	(0.0493)	(0.0465)	(0.0465)
Female	-0.0458	0.0164	0.0350	0.00325	-0.00262
	(0.105)	(0.0284)	(0.0290)	(0.0275)	(0.0273)
Race					
Black	0.166*	0.0478*	0.0346	0.0495*	0.0301
	(0.0959)	(0.0272)	(0.0272)	(0.0260)	(0.0264)
Other race	-0.305*	-0.141***	-0.0686	-0.0448	-0.0677*
	(0.167)	(0.0421)	(0.0462)	(0.0395)	(0.0393)
Hispanic origin	-0.187	0.0184	-0.0458	-0.0837	-0.0108
	(0.332)	(0.0842)	(0.0839)	(0.0692)	(0.0778)
Education					
Less than high school	0.332***	0.0937***	-0.0148	0.0295	0.0407
	(0.113)	(0.0309)	(0.0319)	(0.0296)	(0.0300)
High school	0.372***	0.0984***	-0.0111	0.00647	0.0178
	(0.116)	(0.0319)	(0.0324)	(0.0303)	(0.0303)
Marital status					
Never married	-0.194	-0.125***	0.0232	0.0207	-0.0422
	(0.157)	(0.0422)	(0.0439)	(0.0410)	(0.0407)
Separated or divorced	0.0428	-0.0355	0.108***	0.118***	0.0463
	(0.152)	(0.0391)	(0.0398)	(0.0377)	(0.0378)
Widowed	0.0266	-0.0189	0.0666*	0.0310	0.0219
	(0.146)	(0.0394)	(0.0400)	(0.0366)	(0.0376)

© 2024 Mellor JM et al. *JAMA Health Forum.*

	Plan rating, on scale of 1-10 n=1884	Rated plan a 10 n=1884	Strongly agree: Know who to call about health/ health care choice n=1894	Strongly agree: Confident in understanding of health care system n=1893	Strongly agree: Caring for health/ chronic condition is manageable n=1892
Rural resident	0.161*	0.0418	0.0491*	0.0878***	0.0343
	(0.0926)	(0.0278)	(0.0284)	(0.0277)	(0.0275)
ADL limitations	-0.0323	-0.0149**	0.0103	0.00408	-0.00392
	(0.0225)	(0.00621)	(0.00649)	(0.00605)	(0.00606)
Diagnosed health conditions					
Heart condition	0.111	0.0305	0.00322	0.0197	0.0243
	(0.0865)	(0.0256)	(0.0261)	(0.0244)	(0.0245)
Stroke	-0.188*	-0.0699**	-0.0563*	0.00471	-0.00883
	(0.0994)	(0.0290)	(0.0297)	(0.0278)	(0.0276)
COPD	0.245**	0.0764**	0.0259	-0.00975	0.00426
	(0.102)	(0.0317)	(0.0318)	(0.0297)	(0.0298)
Cancer	0.0216	-0.00508	0.00884	-0.00707	-0.0139
	(0.118)	(0.0315)	(0.0322)	(0.0304)	(0.0301)
Diabetes	-0.0437	-0.00628	0.000185	0.0176	3.56e-06
	(0.0865)	(0.0250)	(0.0255)	(0.0241)	(0.0242)
Asthma	-0.245**	-0.0518*	0.0459	0.0883***	0.0824***
	(0.108)	(0.0286)	(0.0299)	(0.0289)	(0.0284)
Intellectual/Developmental Disability	0.197*	0.0607*	0.00913	-0.0487	-0.00730
	(0.117)	(0.0335)	(0.0348)	(0.0319)	(0.0319)
Depression/Anxiety	-0.298***	-0.0880***	-0.0260	-0.0521**	-0.0599**
	(0.0944)	(0.0258)	(0.0263)	(0.0248)	(0.0249)
Dementia	0.0945	-0.00730	0.0621*	0.0153	0.0340
	(0.106)	(0.0335)	(0.0339)	(0.0314)	(0.0320)
Substance use disorder	-0.596*	-0.116*	-0.214***	-0.106*	-0.141***
	(0.353)	(0.0628)	(0.0612)	(0.0570)	(0.0515)
Another chronic disease	-0.153	-0.0889***	0.00611	-0.0421	-0.0234
	(0.108)	(0.0307)	(0.0326)	(0.0300)	(0.0306)
Constant	8.688***	0.522***	0.344***	0.184***	0.274***
	(0.229)	(0.0635)	(0.0647)	(0.0592)	(0.0596)

NOTES: a) EAE: Exclusively aligned enrollment. *p < 0:10 **p < 0:05 ***p < 0:01

© 2024 Mellor JM et al. *JAMA Health Forum.*

eTable 9: Full Model Results for Dually Eligible Beneficiaries' Experience of Care (models reported in Table 2, Any D-SNP, relative to Traditional Medicare)

	Difficulty with[a]:				
	Primary care n=1885	Specialist care n=1885	Mental health services n=1883	RX medications n=1886	Medical equipment and/or supplies n=1884
Any D-SNP	-0.0188	-0.0114	-0.00633	-0.0306*	-0.0372*
	(0.0162)	(0.0177)	(0.0120)	(0.0178)	(0.0207)
Age in years					
<45	-0.0313	-0.0125	-0.00433	0.00482	0.0953*
	(0.0458)	(0.0495)	(0.0350)	(0.0473)	(0.0536)
45-64	-0.0751**	-0.0650*	-0.0116	-0.0245	0.0369
	(0.0336)	(0.0363)	(0.0266)	(0.0346)	(0.0370)
70-74	-0.0731**	-0.109***	-0.0284	-0.0282	0.0148
	(0.0371)	(0.0383)	(0.0273)	(0.0402)	(0.0422)
75-79	-0.0514	-0.0913**	-0.0346	0.00209	0.0331
	(0.0412)	(0.0405)	(0.0260)	(0.0433)	(0.0405)
80-84	-0.0659*	-0.0771*	-0.0537**	-0.0326	0.0298
	(0.0384)	(0.0399)	(0.0256)	(0.0406)	(0.0407)
85+	-0.0736*	-0.112***	-0.0549**	-0.0485	-0.0111
	(0.0394)	(0.0379)	(0.0250)	(0.0384)	(0.0390)
Female	0.00172	-0.0123	0.00896	-0.00329	-0.0413
	(0.0190)	(0.0208)	(0.0137)	(0.0224)	(0.0253)
Race					
Black	-2.35e-05	-0.0486**	0.00168	-0.0261	-0.0208
	(0.0182)	(0.0193)	(0.0145)	(0.0198)	(0.0238)
Other race	0.0134	0.0567*	0.0448**	-0.0117	-0.0470
	(0.0277)	(0.0327)	(0.0228)	(0.0295)	(0.0324)
Hispanic origin	0.0225	0.0172	-0.0206	-0.0280	0.143*
	(0.0536)	(0.0700)	(0.0243)	(0.0646)	(0.0835)
Education					
Less than high school	0.00259	-0.0376	-0.0127	-0.0231	-0.0685**
	(0.0207)	(0.0241)	(0.0163)	(0.0242)	(0.0275)
High school	0.00741	-0.0279	-0.00805	-0.0222	-0.0368
	(0.0221)	(0.0256)	(0.0178)	(0.0253)	(0.0292)
Marital status					
Never married	-0.0175	-0.0430	0.0148	-0.0542	-0.0500
	(0.0275)	(0.0324)	(0.0181)	(0.0352)	(0.0412)
Separated or divorced	-0.0111	-0.0106	0.0135	0.000986	-0.00653
	(0.0276)	(0.0311)	(0.0192)	(0.0340)	(0.0386)
Widowed	-0.0152	-0.0279	0.00241	-0.0197	-0.0161
	(0.0268)	(0.0288)	(0.0173)	(0.0309)	(0.0343)
Rural resident	-0.0354**	-0.0577***	-0.0205	-0.0331	-0.0415*
	(0.0171)	(0.0185)	(0.0127)	(0.0204)	(0.0238)
ADL limitations	0.0146***	0.0108**	0.00225	0.0131***	0.0353***
	(0.00390)	(0.00434)	(0.00273)	(0.00436)	(0.00481)
Diagnosed health conditions					

© 2024 Mellor JM et al. *JAMA Health Forum.*

	Difficulty with[a]:				
	Primary care n=1885	Specialist care n=1885	Mental health services n=1883	RX medications n=1886	Medical equipment and/or supplies n=1884
Heart condition	0.0353**	0.0152	0.0104	0.0262	0.0173
	(0.0172)	(0.0187)	(0.0133)	(0.0186)	(0.0215)
Stroke	-0.0296	-0.0286	-0.00601	-0.0202	-0.0229
	(0.0185)	(0.0205)	(0.0142)	(0.0212)	(0.0241)
COPD	-0.0145	-0.0413*	-0.0215	-0.0455*	0.00866
	(0.0232)	(0.0241)	(0.0179)	(0.0238)	(0.0300)
Cancer	-0.00546	0.0124	0.0305*	0.0401	-0.0161
	(0.0214)	(0.0227)	(0.0180)	(0.0253)	(0.0262)
Diabetes	-0.0154	-0.0312*	0.00111	0.0259	-0.00774
	(0.0171)	(0.0184)	(0.0133)	(0.0193)	(0.0219)
Asthma	0.0238	0.0295	0.00616	0.0435*	0.0480*
	(0.0212)	(0.0223)	(0.0165)	(0.0222)	(0.0256)
Intellectual/Developmental Disability	-0.0113	-0.0173	-0.00280	0.0182	0.0140
	(0.0229)	(0.0242)	(0.0175)	(0.0273)	(0.0317)
Depression/Anxiety	0.0483***	0.0676***	0.0761***	0.0604***	0.0295
	(0.0177)	(0.0188)	(0.0129)	(0.0202)	(0.0221)
Dementia	-0.0483**	-0.0200	0.00931	-0.0194	-0.0476*
	(0.0208)	(0.0240)	(0.0162)	(0.0232)	(0.0274)
Substance use disorder	0.0994	0.137**	0.0391	0.0635	0.0526
	(0.0639)	(0.0667)	(0.0546)	(0.0595)	(0.0664)
Another chronic disease	0.00669	0.0465*	-0.00241	-0.0128	0.0635**
	(0.0245)	(0.0265)	(0.0181)	(0.0241)	(0.0295)
Constant	0.119***	0.239***	0.0305	0.127**	0.138**
	(0.0459)	(0.0519)	(0.0312)	(0.0511)	(0.0574)

NOTES:

a) Difficulty is defined as having a lot of difficulty or some difficulty, versus no difficulty/no need.

*p < 0:10 **p < 0:05 ***p < 0:01

© 2024 Mellor JM et al. *JAMA Health Forum.*

eTable 9 continued

	Difficulty with[a]:				
	Dental care n=1885	Vision care n=1885	Hearing care n=1880	HCBS[b] n=1886	Any type of care n=1882
Any D-SNP	-0.0158	-0.0127	-0.0204	0.0129	-0.0281
	(0.0205)	(0.0173)	(0.0142)	(0.0195)	(0.0255)
Age in years					
<45	0.191***	0.0738*	0.0178	-0.0550	0.125*
	(0.0588)	(0.0444)	(0.0333)	(0.0469)	(0.0638)
45-64	0.0588	0.0565**	-0.00865	-0.0697*	-0.0166
	(0.0382)	(0.0272)	(0.0226)	(0.0361)	(0.0456)
70-74	-0.00404	0.0766**	-0.00169	-0.0592	-0.0577
	(0.0393)	(0.0325)	(0.0267)	(0.0416)	(0.0514)
75-79	-0.0170	0.0474	0.0312	-0.0452	-0.0178
	(0.0396)	(0.0327)	(0.0281)	(0.0407)	(0.0521)
80-84	0.0120	0.0633**	0.0651**	0.0268	-0.00164
	(0.0415)	(0.0321)	(0.0314)	(0.0451)	(0.0530)
85+	-0.0156	0.0642**	0.0737**	0.000951	0.00887
	(0.0396)	(0.0319)	(0.0301)	(0.0432)	(0.0508)
Female	0.0270	-0.0136	0.00331	-0.00536	-0.0198
	(0.0232)	(0.0204)	(0.0159)	(0.0237)	(0.0302)
Race					
Black	0.00780	0.00723	-0.0125	-0.0266	0.0141
	(0.0229)	(0.0191)	(0.0156)	(0.0214)	(0.0286)
Other race	0.0920**	0.0656**	0.0825***	-0.0274	0.0524
	(0.0359)	(0.0322)	(0.0307)	(0.0337)	(0.0423)
Hispanic origin	0.0183	-0.0117	-0.0545	0.0305	0.0811
	(0.0611)	(0.0494)	(0.0432)	(0.0600)	(0.0858)
Education					
Less than high school	-0.0368	-0.0189	-0.00907	-0.0834***	-0.119***
	(0.0278)	(0.0225)	(0.0190)	(0.0263)	(0.0341)
High school	-0.0337	-0.00865	0.0157	-0.0410	-0.0721**
	(0.0280)	(0.0236)	(0.0195)	(0.0280)	(0.0342)
Marital status					
Never married	0.00197	-0.0482	-0.0197	-0.0328	-0.0603
	(0.0345)	(0.0315)	(0.0218)	(0.0387)	(0.0475)
Separated or divorced	0.0255	-0.00186	0.00918	-0.0527	-0.00888
	(0.0327)	(0.0309)	(0.0230)	(0.0363)	(0.0435)
Widowed	0.0103	0.00690	-0.0111	-0.0396	-0.0151
	(0.0311)	(0.0281)	(0.0236)	(0.0351)	(0.0425)
Rural resident	-0.0555**	-0.0667***	-0.0414***	-0.0353	-0.111***
	(0.0219)	(0.0169)	(0.0137)	(0.0220)	(0.0300)
ADL limitations	0.00790	0.00645	0.00717**	0.0219***	0.0286***
	(0.00516)	(0.00429)	(0.00348)	(0.00468)	(0.00649)
Diagnosed health conditions					
Heart condition	0.0276	0.0168	0.0273*	-0.0240	0.0412
	(0.0217)	(0.0182)	(0.0150)	(0.0207)	(0.0273)

© 2024 Mellor JM et al. *JAMA Health Forum.*

	Difficulty with[a]:				
	Dental care n=1885	Vision care n=1885	Hearing care n=1880	HCBS[b] n=1886	Any type of care n=1882
Stroke	-0.0302	0.00143	-0.00774	-0.00211	-0.0228
	(0.0228)	(0.0212)	(0.0179)	(0.0235)	(0.0301)
COPD	-0.0408	-0.0124	-0.0167	-0.0146	0.00160
	(0.0256)	(0.0254)	(0.0186)	(0.0277)	(0.0345)
Cancer	-0.00159	0.0206	0.0267	-0.0214	0.0231
	(0.0257)	(0.0228)	(0.0218)	(0.0241)	(0.0323)
Diabetes	-0.00551	-0.0322*	0.0131	-0.0234	0.0136
	(0.0220)	(0.0184)	(0.0146)	(0.0202)	(0.0268)
Asthma	0.0569**	0.0493**	0.0195	0.0491**	0.0524*
	(0.0259)	(0.0219)	(0.0186)	(0.0248)	(0.0308)
Intellectual/Developmental Disability	0.0296	0.0409	0.0542**	0.0411	0.0731**
	(0.0307)	(0.0260)	(0.0245)	(0.0312)	(0.0355)
Depression/Anxiety	0.113***	0.0873***	0.0582***	0.0648***	0.153***
	(0.0224)	(0.0179)	(0.0148)	(0.0215)	(0.0282)
Dementia	-0.0517**	-0.0762***	-0.0423**	0.00716	-0.0398
	(0.0257)	(0.0220)	(0.0212)	(0.0291)	(0.0347)
Substance use disorder	0.0670	0.0386	0.0743	0.131*	0.107*
	(0.0716)	(0.0597)	(0.0625)	(0.0708)	(0.0638)
Another chronic disease	0.0351	-0.00117	-0.00790	0.0103	0.0539
	(0.0301)	(0.0238)	(0.0199)	(0.0261)	(0.0341)
Constant	0.0905*	0.0503	0.00819	0.194***	0.376***
	(0.0541)	(0.0457)	(0.0355)	(0.0555)	(0.0677)

NOTES:

a) Difficulty is defined as having a lot of difficulty or some difficulty, versus no difficulty/no need.

b) HCBS (Home and Community Based Services), defined in the survey as "care and other in-home services and conveniences that help with daily activities (personal care services, adult day care, skilled nursing, etc.)

*p < 0:10 **p < 0:05 ***p < 0:01

© 2024 Mellor JM et al. *JAMA Health Forum.*

eTable 9 continued

	Delays getting plan approvals: For medications n=1844	Delays getting plan approvals: For specialist appts n=1841	Delays getting plan approvals: For HCBS[b] n=1846
Any D-SNP	-0.00205	0.0353	0.0159
	(0.0248)	(0.0235)	(0.0253)
Age in years			
<45	-0.0623	-0.0317	-0.121**
	(0.0591)	(0.0619)	(0.0604)
45-64	0.0213	-0.0416	-0.0943**
	(0.0461)	(0.0440)	(0.0467)
70-74	0.0218	-0.0344	-0.0256
	(0.0511)	(0.0493)	(0.0535)
75-79	0.0202	-0.0799	-0.0132
	(0.0545)	(0.0502)	(0.0561)
80-84	-0.0391	-0.0492	0.0243
	(0.0480)	(0.0479)	(0.0523)
85+	-0.0163	-0.0692	-0.0452
	(0.0479)	(0.0471)	(0.0503)
Female	0.0464	0.0326	0.0248
	(0.0292)	(0.0272)	(0.0304)
Race			
Black	0.00400	0.00239	0.0184
	(0.0279)	(0.0265)	(0.0283)
Other race	0.0895**	0.149***	0.0246
	(0.0410)	(0.0407)	(0.0411)
Hispanic origin	0.0634	0.121	0.00953
	(0.0796)	(0.0746)	(0.0759)
Education			
Less than high school	-0.0429	-0.0512	-0.0659**
	(0.0328)	(0.0312)	(0.0331)
High school	-0.0645*	-0.0547*	-0.0287
	(0.0341)	(0.0325)	(0.0347)
Marital status			
Never married	-0.0560	-0.0384	-0.0915**
	(0.0446)	(0.0398)	(0.0463)
Separated or divorced	0.0134	0.00660	-0.0479
	(0.0426)	(0.0383)	(0.0443)
Widowed	-0.0541	0.00181	-0.0726*
	(0.0396)	(0.0360)	(0.0417)
Rural resident	0.0236	0.00869	-0.0133
	(0.0299)	(0.0282)	(0.0309)
ADL limitations	0.0166***	0.00473	0.0188***
	(0.00625)	(0.00615)	(0.00663)
Diagnosed health conditions			

© 2024 Mellor JM et al. *JAMA Health Forum.*

	Delays getting plan approvals: For medications n=1844	Delays getting plan approvals: For specialist appts n=1841	Delays getting plan approvals: For HCBS[b] n=1846
Heart condition	0.0174	0.0491**	0.0206
	(0.0262)	(0.0246)	(0.0266)
Stroke	0.00120	-0.00829	-0.0428
	(0.0287)	(0.0269)	(0.0295)
COPD	-0.0351	-0.0620**	-0.0388
	(0.0338)	(0.0309)	(0.0338)
Cancer	0.0329	0.0204	-0.00164
	(0.0321)	(0.0302)	(0.0322)
Diabetes	0.0853***	0.0147	0.0373
	(0.0262)	(0.0245)	(0.0263)
Asthma	0.119***	0.100***	0.0827***
	(0.0318)	(0.0303)	(0.0315)
Intellectual/Developmental Disability	0.0984***	0.0574*	0.0700**
	(0.0350)	(0.0331)	(0.0343)
Depression/Anxiety	0.0469*	0.0313	0.0174
	(0.0266)	(0.0247)	(0.0270)
Dementia	-0.0462	-0.0352	-0.0449
	(0.0321)	(0.0305)	(0.0333)
Substance use disorder	-0.0246	0.0127	0.0463
	(0.0708)	(0.0736)	(0.0761)
Another chronic disease	0.0314	-0.0451	-0.0155
	(0.0332)	(0.0305)	(0.0336)
Constant	0.157**	0.219***	0.318***
	(0.0640)	(0.0617)	(0.0674)

NOTES:

a) Difficulty is defined as having a lot of difficulty or some difficulty, versus no difficulty/no need.

b) HCBS (Home and Community Based Services), defined in the survey as "care and other in-home services and conveniences that help with daily activities (personal care services, adult day care, skilled nursing, etc.)

*p < 0:10 **p < 0:05 ***p < 0:01

© 2024 Mellor JM et al. *JAMA Health Forum.*

eTable 10: Full Model Results of Dually Eligible Beneficiaries' Out-of-Pocket Spending (for models reported Table 3, Any D-SNP, relative to Traditional Medicare)

	Out-of-Pocket Spending				
	Log (OOP[a] Spending) n=1694	Overall n=1821	Physician Visits n=1913	Prescription drugs n=1913	Dental care n=1913
Any D-SNP	-0.828***	-0.119***	0.0167	-0.0522***	-0.0547***
	(0.158)	(0.0267)	(0.0154)	(0.0202)	(0.0157)
Age in years					
<45	0.0273	-0.0181	0.0524	-0.00648	0.0694
	(0.387)	(0.0682)	(0.0336)	(0.0490)	(0.0470)
45-64	-0.176	-0.0696	0.0242	-0.0194	-0.00708
	(0.277)	(0.0479)	(0.0246)	(0.0396)	(0.0309)
70-74	-0.114	-0.0444	0.0149	-0.0312	-0.00988
	(0.333)	(0.0557)	(0.0340)	(0.0410)	(0.0316)
75-79	-0.135	-0.0453	-0.0242	-0.0565	-0.0627**
	(0.316)	(0.0558)	(0.0236)	(0.0405)	(0.0286)
80-84	-0.0376	-0.0146	-0.0184	-0.0569	-0.0232
	(0.311)	(0.0537)	(0.0250)	(0.0396)	(0.0289)
85+	0.371	0.0852	0.0298	-0.0183	-0.00705
	(0.300)	(0.0528)	(0.0277)	(0.0394)	(0.0286)
Female	0.0686	0.0158	-0.0308	0.00563	0.0158
	(0.187)	(0.0319)	(0.0218)	(0.0243)	(0.0188)
Race					
Black	-0.430**	-0.0616**	0.0125	-0.0138	-0.0517***
	(0.181)	(0.0303)	(0.0166)	(0.0234)	(0.0186)
Other race	-0.0315	0.00232	-0.00168	0.0229	0.0329
	(0.261)	(0.0439)	(0.0235)	(0.0336)	(0.0265)
Hispanic origin	-0.292	0.0387	-0.00419	0.0403	0.0159
	(0.498)	(0.0781)	(0.0446)	(0.0631)	(0.0481)
Education					
Less than high school	-0.693***	-0.117***	-0.0139	-0.00835	-0.0515**
	(0.204)	(0.0346)	(0.0194)	(0.0257)	(0.0210)
High school	-0.281	-0.0427	-0.0315	0.0133	-0.0476**
	(0.217)	(0.0358)	(0.0200)	(0.0293)	(0.0233)
Marital status					
Never married	-0.229	-0.0452	-0.0156	-0.0394	-0.00702
	(0.286)	(0.0493)	(0.0223)	(0.0367)	(0.0286)
Separated or divorced	-0.00448	-0.00442	0.0121	-0.0328	0.0213
	(0.276)	(0.0454)	(0.0250)	(0.0360)	(0.0290)
Widowed	-0.0155	-0.0279	0.0342*	-0.0324	-0.00328
	(0.257)	(0.0435)	(0.0200)	(0.0329)	(0.0242)
Rural resident	-0.171	-0.00450	0.0137	0.0171	0.0201
	(0.181)	(0.0318)	(0.0186)	(0.0250)	(0.0200)
ADL limitations	0.171***	0.0244***	-0.000424	0.00804	-0.00265
	(0.0411)	(0.00701)	(0.00405)	(0.00532)	(0.00452)
Diagnosed health conditions					
Heart condition	0.305*	0.0611**	0.0249	0.0121	0.00284
	(0.167)	(0.0282)	(0.0161)	(0.0210)	(0.0161)

© 2024 Mellor JM et al. *JAMA Health Forum.*

	Out-of-Pocket Spending				
	Log (OOP[a] Spending) n=1694	Overall n=1821	Physician Visits n=1913	Prescription drugs n=1913	Dental care n=1913
Stroke	0.0851	0.0143	-0.0172	0.0349	0.0210
	(0.190)	(0.0319)	(0.0173)	(0.0256)	(0.0196)
COPD	-0.508**	-0.0805**	-0.0340*	-0.0142	-0.0707***
	(0.200)	(0.0350)	(0.0180)	(0.0265)	(0.0177)
Cancer	-0.0947	-0.0254	0.0229	-0.0162	0.00361
	(0.201)	(0.0340)	(0.0210)	(0.0247)	(0.0198)
Diabetes	-0.0519	-0.000268	-0.0127	0.0107	-0.0158
	(0.165)	(0.0278)	(0.0151)	(0.0211)	(0.0169)
Asthma	0.125	0.0219	0.0245	0.0189	0.0393**
	(0.185)	(0.0315)	(0.0189)	(0.0251)	(0.0191)
Intellectual/Developmental Disability	0.0935	0.0343	0.00821	-0.0594**	0.0122
	(0.208)	(0.0362)	(0.0186)	(0.0237)	(0.0199)
Depression/Anxiety	0.419**	0.0792***	0.00652	0.0400*	0.00828
	(0.170)	(0.0290)	(0.0149)	(0.0221)	(0.0173)
Dementia	-0.0461	-0.0380	-0.0113	-0.0275	-0.0184
	(0.214)	(0.0359)	(0.0184)	(0.0247)	(0.0179)
Substance use disorder	0.206	0.0368	0.0165	0.0476	0.00319
	(0.458)	(0.0711)	(0.0424)	(0.0619)	(0.0375)
Another chronic disease	0.748***	0.117***	0.0232	0.0214	0.0101
	(0.229)	(0.0353)	(0.0243)	(0.0287)	(0.0222)
Constant	2.387***	0.490***	0.0702**	0.174***	0.171***
	(0.398)	(0.0690)	(0.0346)	(0.0535)	(0.0421)

NOTES:

a) OOP: out-of-pocket

*p < 0:10 **p < 0:05 ***p < 0:01

© 2024 Mellor JM et al. *JAMA Health Forum.*

eTable 10 continued

	Out-of-Pocket Spending:		
	Vision care n=1913	Medical equipment n=1913	Feel OOP[a] costs are a major financial burden n=1794
Any D-SNP	-0.0179	-0.0399**	-0.0487**
	(0.0146)	(0.0159)	(0.0198)
Age in years			
<45	-0.0145	0.000754	-0.0830*
	(0.0296)	(0.0374)	(0.0491)
45-64	0.0130	-0.0217	-0.0520
	(0.0229)	(0.0289)	(0.0388)
70-74	0.0706**	-0.0516	-0.0573
	(0.0358)	(0.0337)	(0.0440)
75-79	-0.000840	-0.0317	-0.0604
	(0.0230)	(0.0339)	(0.0440)
80-84	-0.0118	-0.0465	-0.0708
	(0.0234)	(0.0335)	(0.0435)
85+	0.0469	-0.0266	-0.0942**
	(0.0288)	(0.0357)	(0.0426)
Female	0.0160	0.0111	0.0200
	(0.0191)	(0.0174)	(0.0231)
Race			
Black	0.00246	-0.0486***	-0.0260
	(0.0161)	(0.0176)	(0.0213)
Other race	0.0261	-0.0331	0.00830
	(0.0240)	(0.0265)	(0.0349)
Hispanic origin	-0.0211	0.0504	0.00392
	(0.0398)	(0.0686)	(0.0709)
Education			
Less than high school	0.0143	-0.0381*	-0.0851***
	(0.0177)	(0.0206)	(0.0269)
High school	0.0204	-0.0103	-0.0393
	(0.0191)	(0.0226)	(0.0283)
Marital status			
Never married	-0.00967	-0.0303	-0.00439
	(0.0218)	(0.0275)	(0.0347)
Separated or divorced	0.00148	0.0125	0.0207
	(0.0226)	(0.0277)	(0.0334)
Widowed	-0.00129	0.00656	0.0199
	(0.0205)	(0.0241)	(0.0312)
Rural resident	-0.00219	-0.0281	-0.0853***
	(0.0173)	(0.0182)	(0.0199)
ADL limitations	-0.000990	0.0192***	0.0204***
	(0.00436)	(0.00356)	(0.00467)
Diagnosed health conditions			
Heart condition	0.0267*	0.0366**	0.0422**

© 2024 Mellor JM et al. *JAMA Health Forum.*

	Out-of-Pocket Spending:		
	Vision care<br>n=1913	Medical equipment<br>n=1913	Feel OOP[a] costs are a major financial burden<br>n=1794
	(0.0147)	(0.0176)	(0.0204)
Stroke	-0.0279*	-0.00504	0.00415
	(0.0149)	(0.0193)	(0.0242)
COPD	-0.0218	-0.0410*	-0.0188
	(0.0175)	(0.0224)	(0.0273)
Cancer	0.000498	-0.00751	0.0428
	(0.0181)	(0.0204)	(0.0272)
Diabetes	-0.00673	-0.00205	-0.0289
	(0.0145)	(0.0172)	(0.0204)
Asthma	0.0270	-0.0107	0.0480*
	(0.0184)	(0.0200)	(0.0257)
Intellectual/Developmental Disability	0.00737	0.0168	0.0102
	(0.0175)	(0.0230)	(0.0281)
Depression/Anxiety	0.0159	0.0157	0.0393*
	(0.0146)	(0.0165)	(0.0220)
Dementia	-0.0350**	-0.00981	-0.0234
	(0.0164)	(0.0235)	(0.0267)
Substance use disorder	-0.0253	-0.00985	0.0827
	(0.0296)	(0.0448)	(0.0724)
Another chronic disease	0.0181	0.0896***	0.0553*
	(0.0237)	(0.0265)	(0.0291)
Constant	0.0348	0.0963**	0.175***
	(0.0290)	(0.0415)	(0.0528)

NOTES:

a) OOP: out-of-pocket

*p < 0:10 **p < 0:05 ***p < 0:01

© 2024 Mellor JM et al. *JAMA Health Forum.*

eTable 11: Full Model Results of Dually Eligible Beneficiaries' Satisfaction (from models reported Table 4, Any D-SNP, relative to Traditional Medicare)

	Very satisfied with:				
	Care Coordinator n=1708	PCP choice n=1871	Specialist choice n=1839	Customer service: Always gives needed info/help n=1080	Customer service: Always treats with courtesy/ respect n=1078
Any D-SNP	-0.00277	0.00698	-0.0177	0.0354	0.0323
	(0.0259)	(0.0266)	(0.0270)	(0.0353)	(0.0286)
Age in years					
<45	-0.101	-0.166**	-0.109	-0.137	-0.0504
	(0.0694)	(0.0665)	(0.0689)	(0.0889)	(0.0840)
45-64	0.00564	-0.00558	0.0217	0.108*	0.0777*
	(0.0447)	(0.0468)	(0.0480)	(0.0607)	(0.0419)
70-74	-0.0429	-0.00121	0.0114	-0.0263	-0.0324
	(0.0511)	(0.0524)	(0.0532)	(0.0684)	(0.0563)
75-79	-0.0365	-0.0920	-0.0766	0.0257	0.0160
	(0.0497)	(0.0561)	(0.0563)	(0.0734)	(0.0650)
80-84	-0.122**	0.00212	0.000822	-0.0594	-0.0793
	(0.0522)	(0.0523)	(0.0526)	(0.0678)	(0.0598)
85+	-0.0822*	-0.0819	-0.0992*	0.0207	-0.0409
	(0.0495)	(0.0523)	(0.0526)	(0.0682)	(0.0562)
Female	0.0288	0.0242	0.0369	0.0188	-0.0113
	(0.0320)	(0.0317)	(0.0319)	(0.0412)	(0.0338)
Race					
Black	0.0360	0.0465	0.0303	0.0279	-0.000990
	(0.0294)	(0.0298)	(0.0302)	(0.0403)	(0.0299)
Other race	-0.0573	-0.0300	-0.0643	-0.231***	-0.311***
	(0.0452)	(0.0438)	(0.0440)	(0.0525)	(0.0542)
Hispanic origin	-0.0141	0.0658	0.0182	-0.0304	-0.0685
	(0.0883)	(0.0783)	(0.0792)	(0.0909)	(0.0968)
Education					
Less than high school	0.131***	-0.0289	0.00110	0.0775*	0.0842**
	(0.0344)	(0.0349)	(0.0354)	(0.0436)	(0.0374)
High school	0.0603	0.0381	0.0545	0.0755	0.0587*
	(0.0368)	(0.0347)	(0.0350)	(0.0460)	(0.0356)
Marital status					
Never married	-0.0191	-0.0963**	-0.104**	0.0392	0.00916
	(0.0472)	(0.0471)	(0.0476)	(0.0619)	(0.0516)
Separated or divorced	0.000850	-0.0945**	-0.103**	0.0830	-0.0210
	(0.0447)	(0.0448)	(0.0453)	(0.0593)	(0.0445)
Widowed	0.00837	-0.0320	-0.0450	0.0502	-0.0171
	(0.0437)	(0.0429)	(0.0432)	(0.0565)	(0.0478)
Rural resident	0.0845***	0.0115	0.00464	0.0507	0.00133
	(0.0294)	(0.0312)	(0.0317)	(0.0426)	(0.0313)
ADL limitations	-0.0148**	-0.0164**	-0.0127*	0.00993	-0.00405

© 2024 Mellor JM et al. *JAMA Health Forum.*

	Very satisfied with:				
	Care Coordinator n=1708	PCP choice n=1871	Specialist choice n=1839	Customer service: Always gives needed info/help n=1080	Customer service: Always treats with courtesy/ respect n=1078
	(0.00675)	(0.00685)	(0.00700)	(0.00931)	(0.00693)
Diagnosed health conditions					
Heart condition	0.0350	0.0141	0.0318	0.0183	-0.00357
	(0.0280)	(0.0281)	(0.0284)	(0.0368)	(0.0295)
Stroke	-0.0426	-0.0223	0.00268	-0.0169	-0.00112
	(0.0316)	(0.0310)	(0.0316)	(0.0432)	(0.0323)
COPD	-0.00444	0.0455	0.0617*	0.0176	-0.00251
	(0.0348)	(0.0338)	(0.0341)	(0.0446)	(0.0354)
Cancer	-0.0203	-0.0313	-0.0419	-0.0192	-0.0805**
	(0.0339)	(0.0335)	(0.0340)	(0.0453)	(0.0402)
Diabetes	-0.0517*	-0.0210	-0.0121	-0.0784**	-0.0152
	(0.0269)	(0.0276)	(0.0282)	(0.0359)	(0.0279)
Asthma	0.0400	0.0260	0.0258	-0.0250	0.00983
	(0.0309)	(0.0305)	(0.0307)	(0.0393)	(0.0309)
Intellectual/Developmental Disability	-0.0280	0.0645*	0.0374	0.106**	-0.0512
	(0.0358)	(0.0374)	(0.0377)	(0.0478)	(0.0408)
Depression/Anxiety	-0.0636**	-0.0696**	-0.0856***	-0.0522	0.0511*
	(0.0279)	(0.0286)	(0.0287)	(0.0379)	(0.0295)
Dementia	0.0414	-0.00990	0.0127	-0.0543	-0.0315
	(0.0348)	(0.0357)	(0.0365)	(0.0462)	(0.0401)
Substance use disorder	-0.145*	-0.170**	-0.185**	-0.123	-0.165**
	(0.0843)	(0.0728)	(0.0743)	(0.0882)	(0.0793)
Another chronic disease	-0.0319	-0.0259	-0.0566	-0.0451	-0.00102
	(0.0359)	(0.0361)	(0.0363)	(0.0476)	(0.0353)
Constant	0.698***	0.742***	0.691***	0.321***	0.800***
	(0.0670)	(0.0672)	(0.0676)	(0.0944)	(0.0687)

*p < 0:10 **p < 0:05 ***p < 0:01

© 2024 Mellor JM et al. *JAMA Health Forum.*

eTable 11 continued

	Plan rating, on scale of 1-10 n=1884	Rated plan a 10 n=1884	Strongly agree: Know who to call about health/ health care n=1894	Strongly agree: Confident in understanding of health care system n=1893	Strongly agree: Caring for health/ chronic condition is manageable n=1892
Any D-SNP	0.199**	0.0592**	0.0134	0.0392	0.0376
	(0.0920)	(0.0259)	(0.0266)	(0.0247)	(0.0250)
Age in years					
<45	-0.892***	-0.176***	-0.0172	0.0166	0.0410
	(0.237)	(0.0637)	(0.0674)	(0.0631)	(0.0633)
45-64	-0.105	-0.0272	0.0275	0.0559	0.0363
	(0.170)	(0.0476)	(0.0484)	(0.0457)	(0.0447)
70-74	-0.0259	0.0142	-0.0391	0.0380	0.0510
	(0.162)	(0.0534)	(0.0547)	(0.0522)	(0.0512)
75-79	-0.0590	-0.0295	-0.0515	-0.0259	-0.0553
	(0.173)	(0.0551)	(0.0561)	(0.0520)	(0.0509)
80-84	-0.437**	-0.112**	-0.0877	-0.0536	-0.0217
	(0.188)	(0.0528)	(0.0537)	(0.0492)	(0.0498)
85+	-0.236	-0.136***	-0.141***	-0.0593	-0.0598
	(0.172)	(0.0511)	(0.0519)	(0.0491)	(0.0490)
Female	-0.0152	0.0129	0.0270	-0.00449	-0.0109
	(0.118)	(0.0305)	(0.0314)	(0.0301)	(0.0302)
Race					
Black	0.148	0.0479	0.0359	0.0410	0.0235
	(0.107)	(0.0299)	(0.0302)	(0.0281)	(0.0287)
Other race	-0.242*	-0.127***	-0.0620	-0.0206	-0.0455
	(0.144)	(0.0396)	(0.0421)	(0.0378)	(0.0386)
Hispanic origin	-0.0143	0.0337	0.0233	-0.0466	0.0131
	(0.253)	(0.0832)	(0.0888)	(0.0779)	(0.0815)
Education					
Less than high school	0.292**	0.0990***	-0.00673	0.0446	0.0479
	(0.129)	(0.0335)	(0.0338)	(0.0317)	(0.0324)
High school	0.278**	0.0830**	-0.0184	0.00125	0.0125
	(0.130)	(0.0347)	(0.0354)	(0.0328)	(0.0329)
Never married	-0.123	-0.118***	0.0355	0.0217	-0.0337
	(0.156)	(0.0456)	(0.0469)	(0.0438)	(0.0441)
Separated or divorced	0.00447	-0.0598	0.108**	0.126***	0.0573
	(0.164)	(0.0436)	(0.0445)	(0.0418)	(0.0421)
Widowed	-0.0154	-0.0427	0.0762*	0.00640	0.00737
	(0.152)	(0.0412)	(0.0420)	(0.0385)	(0.0397)
Rural resident	0.166	0.0467	0.0644**	0.101***	0.0364
	(0.109)	(0.0310)	(0.0319)	(0.0307)	(0.0305)

© 2024 Mellor JM et al. *JAMA Health Forum.*

	Plan rating, on scale of 1-10<br>n=1884	Rated plan a 10<br>n=1884	Strongly agree: Know who to call about health/ health care<br>n=1894	Strongly agree: Confident in understanding of health care system<br>n=1893	Strongly agree: Caring for health/ chronic condition is manageable<br>n=1892
ADL limitations	-0.0269	-0.0159**	0.00544	0.00107	-0.00410
	(0.0246)	(0.00679)	(0.00702)	(0.00659)	(0.00657)
Diagnosed health conditions					
Heart condition	0.0924	0.0358	0.0163	0.0247	0.0459*
	(0.0951)	(0.0273)	(0.0280)	(0.0260)	(0.0264)
Stroke	-0.221**	-0.0855***	-0.0718**	-0.00981	-0.0252
	(0.109)	(0.0311)	(0.0319)	(0.0294)	(0.0293)
COPD	0.279**	0.0904***	0.0130	-0.0127	-0.00745
	(0.113)	(0.0345)	(0.0352)	(0.0328)	(0.0324)
Cancer	0.0547	-0.0131	-0.0155	-0.0279	-0.0376
	(0.113)	(0.0332)	(0.0346)	(0.0320)	(0.0315)
Diabetes	-0.0312	-0.00383	0.00517	0.0165	0.00217
	(0.0906)	(0.0271)	(0.0278)	(0.0258)	(0.0262)
Asthma	-0.212*	-0.0407	0.0495	0.0962***	0.0862***
	(0.111)	(0.0306)	(0.0318)	(0.0304)	(0.0302)
Intellectual/Developmental Disability	0.208	0.0628*	-0.0299	-0.0695**	-0.0160
	(0.127)	(0.0344)	(0.0362)	(0.0329)	(0.0337)
Depression/Anxiety	-0.340***	-0.0906***	-0.0177	-0.0669**	-0.0625**
	(0.101)	(0.0276)	(0.0281)	(0.0262)	(0.0267)
Dementia	0.00122	-0.0285	0.0750**	0.0253	0.0283
	(0.113)	(0.0336)	(0.0350)	(0.0321)	(0.0325)
Substance use disorder	-0.674	-0.0979	-0.213***	-0.0850	-0.145**
	(0.453)	(0.0657)	(0.0683)	(0.0647)	(0.0571)
Another chronic disease	-0.175	-0.109***	-0.00157	-0.0637**	-0.0308
	(0.113)	(0.0334)	(0.0357)	(0.0316)	(0.0331)
Constant	8.762***	0.549***	0.379***	0.230***	0.282***
	(0.246)	(0.0683)	(0.0686)	(0.0613)	(0.0620)

*p < 0:10 **p < 0:05 ***p < 0:01

© 2024 Mellor JM et al. *JAMA Health Forum.*
