# Supplementary material for: Beneficiary Experience of Care by Level of Integration in Dual Eligible Special Needs Plans
Source: JAMA Health Forum. 2024 Jun 7;5(6):e241383. doi: 10.1001/jamahealthforum.2024.1383 (PMC11161838; doi:10.1001/jamahealthforum.2024.1383)
Supplement: Supplement 2. — Data Sharing Statement [file jamahealthforum-e241383-s002.pdf]

Data Sharing Statement

Mellor. Beneficiary Experience of Care by Level of Integration in Dual Eligible Special Needs Plans. *JAMA Health Forum*. Published June 07, 2024.
doi:10.1001/jamahealthforum.2024.1383

Data
Data available: No

Additional Information
Explanation for why data not available: The survey data used for this study cannot be shared due to a Data Use Agreement with the Virginia Department of Medical Assistance Services (the funder) that prevents public release of data used for the evaluation of Commonwealth Coordinated Care Plus in order to protect the confidentiality of Medicaid members who participated.
